# Chitin/Graphene Oxide Composite Materials for Heavy-Metal-Ion Adsorption

**DOI:** 10.1021/acsomega.6c00814

**Published:** 2026-05-21

**Authors:** Anjana Aravind, Kevin Gerein, Andreas Hirsch, Frank Hauke, Eike Brunner

**Affiliations:** † Chair of Bioanalytical Chemistry, Faculty of Chemistry and Food Chemistry, TU Dresden, Dresden 01069, Germany; ‡ Chair of Organic Chemistry II, Department of Chemistry and Pharmacy, FAU Erlangen-Nürnberg, Erlangen 91058, Germany

## Abstract

Rare earth elements (REEs) are vital for modern technology owing to their unique properties and widespread industrial applications. However, their growing use results in increased environmental releases, raising concerns about potential ecological impacts and human health risks. The same is true for actinides like uranium. Efficient removal of these elements from water, particularly in the mining and nuclear industry, is thus crucial. Recycling these elements has emerged as a sustainable approach for wastewater processing and waste reuse. Adsorption presents advantageous properties for effective contaminant removal. The present study focuses on the synthesis and characterization of composite adsorbent materials for this application. Chitin is an abundant biopolymer with particularly promising properties, including widespread availability, low cost, biocompatibility, and biodegradability. However, the processing of chitin into stable materials is challenging due to its very low solubility. Previously, we reported the cross-linking of α-chitin with its monomer by processing in the ionic liquid (IL) 1-butyl-3-methylimidazolium acetate. These composites, however, exhibit a lower adsorption capacity than pure chitin powder due to partial blocking of adsorption sites. To overcome this problem, we have now incorporated graphene oxide (GO) into the chitin-based composite. With its large surface area and various functional groups, GO further enhances the inherent properties of chitin. These composites are shown to very efficiently remove even spurious amounts of europium and uranium from aqueous solutions.

## Graphical abstract

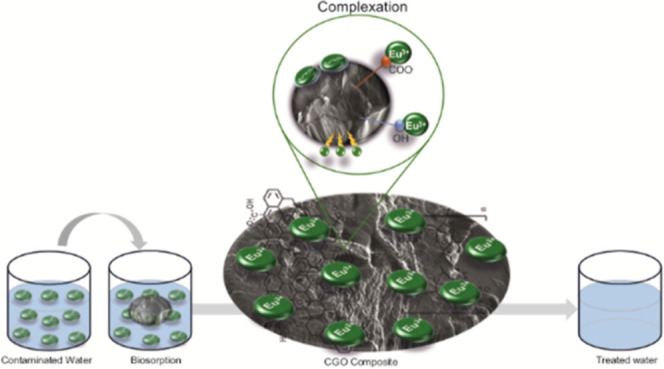

## 1 Introduction

Water quality is crucial for the stability of aquatic and terrestrial ecosystems as well as human health. Likewise, water is a fundamental resource for many industrial processes.^1,2^ However, industrialization leads to the release of hazardous byproducts, including heavy metals—often linked to environmental challenges. Continuous exposure to even low concentrations of heavy metals poses significant health risks due to their bioaccumulation and enrichment in the food chain.^3^ Recovery of these substances from contaminated water would be an attractive solution to this problem as heavy metals are valuable materials for various industrial purposes. Ideally, contaminants would be removed from waste before their release into the environment and recovered for further use.^4^ Chitin (C_8_H_13_O_5_N)_n_ is a low-cost biopolymer and well-known for its favorable adsorption properties of metal ions from solutions, including uranium.^5–9^ This is due to its chemical functionalities, especially carbonyl and hydroxyl groups. Chitin is the second most abundant biopolymer on earth after cellulose.^10–14^ This polysaccharide is formed by linearly β-1,4-linked *N*-acetyl- *d* -glucosamine (GlcNAc) units.^15,16^ Chitin is a component of fungal cell walls and also found in the exoskeletons of numerous invertebrates, such as crustaceans and insect cuticles.^15,16^ It is also present in the skeletons of some marine sponges.^17,18^ Chitin is also produced by several other living organisms in the lower plant and animal kingdoms, serving many functions, especially protection by providing mechanical strength and reinforcement.^19^ It is well-suited for various applications due to its excellent biocompatibility, biodegradability, hydrophilicity, and low toxicity and is readily available from renewable sources.^20–28^ Depending on its source, chitin occurs in three allomorphs: α, β, and γ. These forms can be differentiated by infrared (IR) and solid-state nuclear magnetic resonance (NMR) spectroscopy as well as X-ray diffraction.^29^ α-Chitin is the most stable form. However, α-chitin cannot swell, and its specific surface area is rather low. Most functional groups are thus inaccessible for metal ions. Using a unique, sponge-like^5^ form of α-chitin extracted from a marine sponge with a comparably high accessible surface area, increasing adsorption capacities for uranyl species from aqueous solutions were observed. However, the primary sources of chitin are crab and shrimp shells. This material is commercially available as a powder consisting of small, compact α-chitin particles. Processing this commercially available material into stable, self-supporting forms such as membranes would be highly desirable. However, due to its stable, strongly hydrogen-bonded structure, chitin is hardly soluble in common organic solvents or in water.^30^ Dissolution of chitin is only feasible in very few solvents, which are usually toxic, degradable, corrosive, scarce, or mutagenic. Therefore, most current uses of this natural resource involve chitosan, a soluble, partially or fully deacetylated derivative of chitin. Various chitosan- and chitin-based composite systems were developed for heavy metal adsorption, including chitosan–PMA–halloysite nanotube composites,^31^ biomimetic SiO_2_@chitosan composites,^32^ magnetite chitosan composites,^33–39^ chemically modified chitin nanocomposites,^40^ yeast-ornamented chitin nanofiber aerogels,^41^ and mineral-based chitosan composites^42^ or di-(2-ethylhexyl) phosphoric acid (D2EHPA)-impregnated chitin composites.^43^ Taken together, these studies demonstrate the continuing interest and innovation in the design of chitin-based composites for heavy metal adsorption purposes.

Previously, we developed an α-chitin dissolution approach in the ionic liquid (IL) 1-butyl-3-methylimidazolium acetate based on the work of Wu et al.^44^ and cross-linked it with the added monomer GlcNAc,^45^ yielding stable composites. However, the cross-linking reaction results in reduced adsorption capacities compared to that of the initial chitin powder as it especially blocks C=O groups, which are tentative interaction sites for heavy metal adsorption.

Graphene oxide (GO) is a layered carbon material with oxygen-containing functional groups attached to both sides of the layer as well as the edges of the plane.^46^ GO consists of a distorted basal plane of carbon atoms with significantly disrupted sp^2^ hybridization due to the covalent attachment of various oxygen-containing functional groups, including hydroxyl (OH), epoxy (ether bridges, C–O–C), carbonyl (C=O), and carboxyl (COOH) groups. These oxygen-containing functional groups disrupt the strong π–π stacking interactions and the regular ABAB stacking order in graphite, leading to a significantly increased interlayer spacing due to electrostatic repulsion between the oxygen functionalities and the intercalation of water molecules. The presence of these oxygen-containing groups makes GO highly hydrophilic, allowing it to disperse readily in water and organic solvents. Owing to its large theoretical specific surface area of 2630 m^2^g^–1^ and abundant functional groups,^47^ GO has proven to be a promising adsorbent. Adsorbents can be designed flexibly in various forms like hydrogels,^48^ membranes,^49^ particles,^50^ sponges, or foams.^51,52^ GO was demonstrated to be a well-suited material for effectively adsorbing heavy metal ions such as lead Pb (II),^53^ cobalt Co (II),^54^ copper Cu­(II),^55^ mercury Hg­(II),^56^ cadmium Cd­(II),^57^ and chromium Cr­(VI).^58^ The only major drawback of GO is that it usually suffers from severe agglomeration, leading to considerably reduced adsorption capacities.^59^ Strong interfacial interactions between graphene oxide sheets lead to agglomeration and limited dispersion in aqueous media, thereby decreasing surface reactivity and accessible surface area. This limits the adsorption performance and thus applications in wastewater treatment.^60^ Moreover, it is a relatively expensive high-end material. To overcome these limitations and harness the complementary properties of chitin and graphene oxide, we here investigate the feasibility of synthesizing chitin/GO (CGO) composite materials, which are expected to exhibit greater stability and enhanced adsorption properties compared to the individual components. Based on our previous research, the employed synthesis procedure relies on dissolution facilitated by ionic liquids.

To evaluate the adsorption properties of the synthesized CGO composites, we studied the adsorption of trivalent europium (Eu­(III)) and hexavalent uranium (U­(VI)) ions. Europium is commonly used as a homologue for trivalent lanthanides and actinides due to its comparable physicochemical properties, whereas uranium is used as a homologue for hexavalent actinides due to its occurrence in nature and mining industries.^61^ For quantification, we used inductively coupled plasma optical emission spectroscopy (ICP-OES). The morphology, structure, and surface properties of the obtained materials were characterized by Fourier transform infrared (FTIR) spectroscopy, solid-state NMR spectroscopy, and scanning electron microscopy (SEM). Various parameters that potentially influence the adsorption efficiency are discussed in detail—including contact time, adsorbent mass, solution pH, and initial ion concentration.

## 2 Materials and Methods

### 2.1 Materials

α-Chitin from crab shells was purchased from Carl Roth GmbH + Co. KG (400 000 g/mol molecular mass). 1-Butyl-3-methyl-imidazolium acetate [BMIM]­[OAc] (≥98% purity) was purchased from IoLiTech-Ionic Liquids Technologies GmbH (Germany). Graphite (Grade A625) was obtained from Asbury Graphite Mills Inc. (Lot: 7585/1). All other chemicals were purchased from Sigma-Aldrich Chemie GmbH and used without further purification. HPLC-grade ethanol and acetone were used.

Graphene oxide was synthesized according to a modified Hummers procedure.^62^ 1.0 g of graphite powder and 0.5 g of sodium nitrate were dispersed in concentrated sulfuric acid (23 mL) while cooling in an ice bath and stirring for 4 h. Subsequently, 3.0 g of potassium permanganate was added, and the mixture heated to 35 °C for 2 h. Afterward, the mixture was carefully diluted with 46 mL of deionized water. After further diluting the mixture with 100 mL of water, it was stirred at 98 °C for 2 h. To reduce residual potassium permanganate and other manganese species, 10 mL of 30% hydrogen peroxide solution was added. Subsequently, the mixture was centrifuged at 15 000 rpm (RCF 21457) for 30 min. The supernatant was removed, and the residue was washed five times by redispersing in deionized water and centrifuged afterward. After washing, the brown precipitate slurry was dried in the oven at 88 °C for several days. The composite materials synthesis procedure is schematically described in Figure 1.

**Figure 1:**
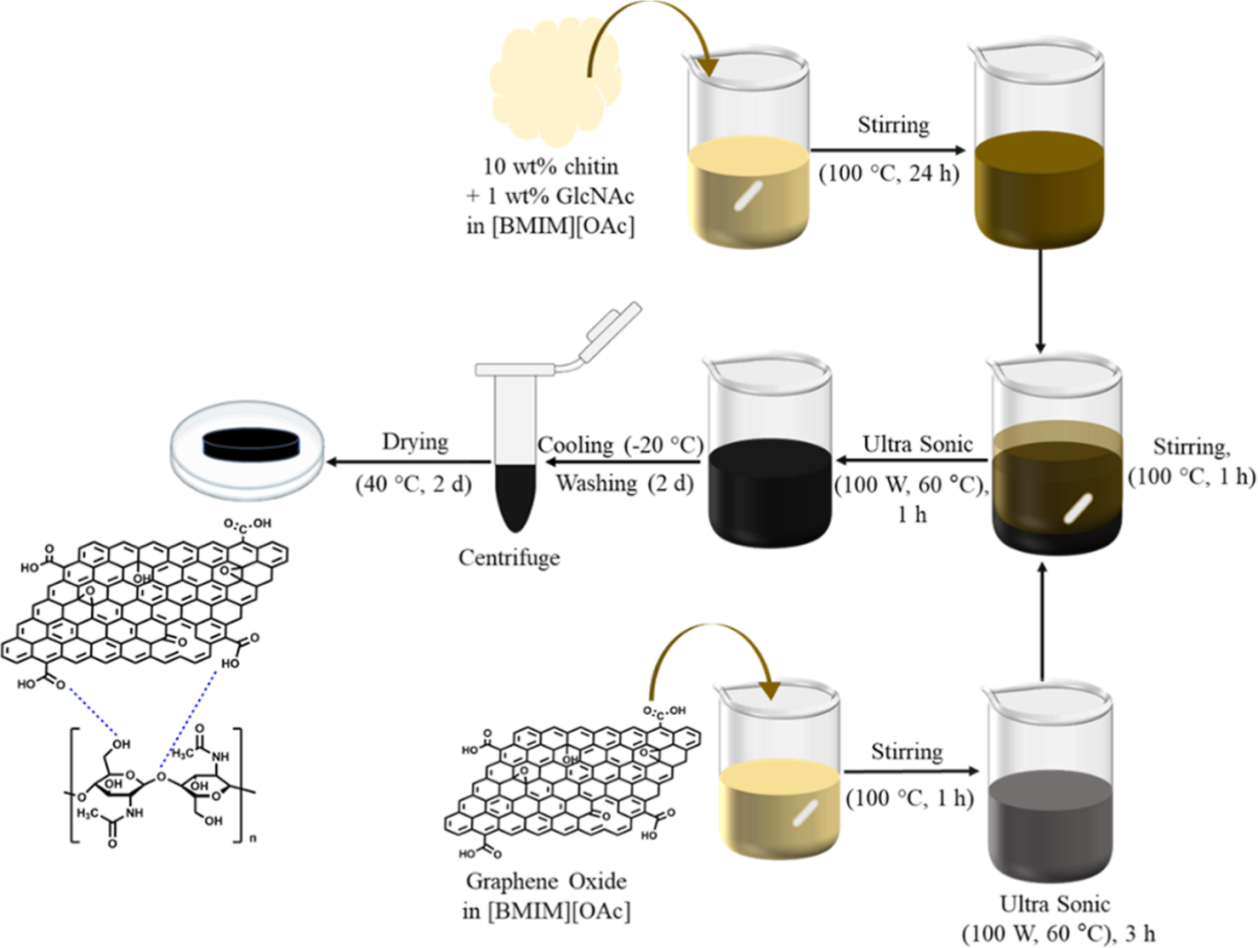
Experimental scheme for the CGO composite synthesis procedure.

It involves chitin dissolution in IL [BMIM]­[OAc],^44,63^ followed by the addition of monomer GlcNAc and graphene oxide. The solution was prepared by dissolving 0.3 g of chitin and 0.03 g of GlcNAc in 3 mL of [BMIM]­[OAc]. Upon heating and stirring, the solution gradually developed an amber coloration. In parallel, 0.15 g of GO was suspended in 3 mL of [BMIM]­[OAc] and stirred at 100 °C for 60 min. Afterward, the solution was transferred to an ultrasonic bath (100 W, 60 °C) for 3 h. Different GO/IL and chitin/IL ratios were mixed to systematically vary the chitin-to-GO ratio. A sample series was synthesized with chitin/GO mass ratios between 1:1 and 7:1 (1:1, 2:1, 3:1, 4:1, 5:1, and 7:1) to evaluate the effect of GO content on the composite structure and properties. Afterward, the mixture was treated in an ultrasonic bath (100 W, 60 °C) for 60 min. The hot solution was poured onto a glass plate and cooled to room temperature. The mixture was cooled overnight at −20 °C prior to washing, which supports network formation. Ionic liquids disrupt the native hydrogen-bonding network of chitin, resulting in highly mobile polymer chains. Gradual cooling promotes re-establishment of hydrogen bonding and structural consolidation of the CGO composites. This stabilization process minimizes structural collapse, GO restacking, and pore shrinkage during subsequent solvent exchange with ethanol and acetone, while washing allows more effective removal of the residual IL. Afterward, the regenerated composite was centrifuged at 5000 rpm for 15 min and then dried in a drying cabinet at 40 °C for 2 days.

### 2.2 Adsorption Experiments

The composites were studied in batch adsorption experiments from solutions containing Eu­(III) and U­(VI). The CGO composite was ground in an agate mortar into powder prior to adsorption experiments to enhance surface area and ensure homogeneous dispersion in aqueous solution, as well as the contact between the adsorbent and metal ions.

Seven types of adsorption experiments were conducted: (i) adsorption performance evaluation of the CGO 1:1 composite, (ii) adsorption performance evaluation of the CGO 7:1 composite, (iii) kinetics studies as a function of contact time for CGO 7:1, (iv) adsorption performance at variable adsorbent mass, (v) adsorption performance at different pH values, (vi) adsorption performance at different initial Eu­(III) ion concentrations, and (vii) desorption experiments. Experiments were performed in triplicate under identical conditions to ensure reproducibility. The reported data are mean values of the three independent measurements. Errors were estimated for 95.4% statistical certainty from the standard deviation Δσ of the three measurements as follows: Δσ times Student́’s *t*-factor divided by the square root of 3 = Δσ × 4.3/1.73 = 1.48 × Δσ.

The first adsorption studies were conducted with an initial concentration of 10^–5^ mol/L Eu­(III) and U­(VI) and 15 mg (±0.3 mg) of the adsorbent (chitin, GO, and the CGO composite) at a temperature of 25 °C and an agitation speed of 200 rpm. Each adsorbent aliquot was suspended in 2 mL of solution. In Batch I, the adsorbents (CGO 1:1, CGO 7:1, GO, and chitin) were shaken in total for 7 days in 10^–5^ mol/L EuCl_3_^•^6H_2_O in ultrapure water. In Batch II, the adsorbents (CGO 7:1, GO, and chitin) were shaken for 7 days in a solution containing 10^–5^ mol/L UO_2_(NO_3_)_2_ dissolved in ultrapure water. An aliquot was taken after 24 h from both batches. Afterward, the samples were centrifuged at 5000 rpm for 10 min. The supernatant was separated from the pellet and analyzed by ICP-OES. The sample pellet was dried under ambient conditions. From this, the adsorption capacity (*q*_*t*_) was calculated as follows^58^

1 $$q_{t}=\frac{\left(C_{O}-C_{t}\right)\times V}{m}$$

where *C*_0_ and *C*_*t*_ (mg L^–1^) are the ion concentrations in the solution before and after adsorption, respectively; *V* is the volume of the solution; and *m* is the weight of the adsorbent.

To determine adsorption kinetics, the adsorption capacity of CGO was measured under contact time variation. Samples were brought into contact with an ion-containing solution for different durations: 30 min, 1, 2, 3, 4, 6, 7, 8, 10, 12, 14, 18, 20, 22, and 24 h.

Adsorption capacities *q*_*t*_ (see eq 1) and removal efficiencies in percent (%R) were then calculated. %R was determined according to eq 2.^64^

2 $$\%R=\eta =\frac{C_{O}-C_{t}}{C_{O}}\times 100\%$$

Kinetic modeling was employed for fitting the data to a pseudo-first-order kinetic model^65,66^ (eq 3) and a pseudo-second-order kinetic model^65,66^ (eq 4)­

3 $$\ln\left(q_{e}-q_{t}\right)=\ln q_{e}-k_{1}t$$

4 $$\frac{t}{q_{t}}=\frac{1}{{{k_{2}q}_{e}}^{2}}+\frac{t}{q_{e}}$$

where *q*_e_ (mg g^–1^) is the adsorption capacity at equilibrium and *q*_*t*_ (mg g^–1^) is the adsorption capacity at a specific time *t* (h). *k*_1_ (h^–1^) and *k*_2_ (mg^–1^ g h^–1^) are the adsorption rate constants for the pseudo-first-order and the pseudo-second-order kinetic model, respectively.

To determine the adsorption isotherm, the adsorption capacity of CGO was evaluated at equilibrium for different initial Eu­(III) concentrations. The adsorption equilibrium of Eu­(III) on the CGO composite was analyzed using the Langmuir isotherm model, which better describes the data than the Freundlich isotherm model (cf. ESI, Figure S6). The Langmuir isotherm and its linearized form are expressed by eqs 5 and 6.^67,68^

5 $${\begin{array}{l} q \end{array}}_{e}=\frac{q_{m}K_{L}C_{e}}{1+K_{L}C_{e}}$$

6 $$\frac{C_{e}}{q_{e}}=\frac{C_{e}}{q_{m}}+\frac{1}{{K_{L}q}_{m}}$$

*C*_e_ (mg L^–1^) is the equilibrium Eu­(III) concentration in solution, *q*_e_ (mg g^–1^) is the the adsorption capacity at equilibrium, *q*_*m*_ (mg g^–1^) is the maximum monolayer adsorption capacity, and *K*_*L*_ (L mg^–1^) is the Langmuir adsorption constant related to the affinity of binding sites. A plot of $\frac{C_{e}}{q_{e}}$ versus *C*_e_ gives a linear function. Its slope and intercept correspond to $\frac{1}{q_{m}}$ and $\frac{1}{{K_{L}q}_{m}}$ , respectively.

After preparation of the aqueous metal-ion solutions, the initial pH was approximately 6.1 for uranium and 5.1 for europium. These values were used directly in the adsorption experiments. No visible precipitation was observed under these experimental conditions, indicating that metal removal occurred primarily through adsorption rather than bulk hydroxide formation. In addition, the pH of the solutions remained essentially unchanged after adsorption, i.e., adsorption did not significantly affect the solution acidity. The pH was adjusted only during the pH-dependent adsorption experiments by using 0.01 M HNO_3_ to obtain the desired pH values.

To analyze the sorption properties of the CGO composites and pure chitin, the supernatant from the batch adsorption tests was analyzed by ICP-OES and compared to the initial concentration of the Eu­(III) and U­(VI) solution. Samples were dissolved in HNO_3_ (5%) and analyzed using an Optima 7000DV spectrometer with the following parameters: high-frequency power: 1300 W; liquid flow: 1.6 L/min; plasma gas flow: 15 L/min; auxiliary gas flow: 0.2 L/min; and nebulizer gas flow: 0.65 L/min. The spectral line at 412.970 nm was used for the radial detection of europium. The spectral line at 385.958 nm was used for the radial detection of uranium.

### 2.3 Desorption Experiments

The adsorbent was first incubated in 10 mL of Eu­(III) solution (10^–5^ M) and stirred at 5000 rpm at room temperature (25 °C) for 24 h for equilibration. The solution was then separated from the composite by centrifugation, and the adsorbent was dried overnight. The amount of adsorbed Eu­(III) was determined using the same analytical method as that employed in the adsorption experiments. The loaded adsorbent was subsequently treated with 10 mL of nitric acid (0.05 and 0.2 M HNO_3_) for 24 h under constant stirring to desorb the metal ions. After desorption, the adsorbent was washed four times with ultrapure water, and the adsorption–desorption cycle was repeated. The percentage of desorbed Eu­(III) was calculated using the following equation^69^

7 $$desorption=\frac{C_{des}\times V_{des}}{\left(C_{O}-C_{e}\right)\times V}\times 100$$

*C*_des_: concentration of Eu­(III) in the desorption solution (mg L^–1^); *V*_des_: volume of desorption solution (L); *C*_O_: initial concentration (mg L^–1^); *C*_e_: equilibrium concentration after adsorption (mg L^–1^); and *V*: volume of adsorption solution (L).

### 2.4 Sample Characterization Techniques

Scanning electron microscopy (SEM) was employed to analyze the morphologies of the chitin and CGO composites. Surface morphology was examined at room temperature using an Oxford XMaxN 150 scanning electron microscope with a 150 mm^2^ detector area and an acceleration voltage of 50 kV at the Dresden Center for Nanoanalysis (DCN), TU Dresden.

Fourier transform infrared (FTIR) spectroscopy was conducted using a Thermo Scientific Nicolet iS5 spectrometer with a broad-band mercury–cadmium–telluride detector. Spectra were acquired in the attenuated total reflection (ATR) mode with a single-reflection monolithic diamond ATR Specac Golden Gate accessory. Each spectrum was recorded from a 2 mm diameter area of the sample in contact with the diamond crystal. Data were collected over the 4000–400 cm^–1^ range, averaging 500 scans at 2 cm^–1^ resolution.

Solid-state ^13^C NMR spectra were acquired using a Bruker Ascend 300 MHz NMR spectrometer operating at 75.47 MHz for ^13^C. A commercial double-resonance 2.5 mm magic-angle spinning (MAS) NMR probe was employed. Approximately 5 mg of the sample was packed into rotors and spun at 15 kHz. Cross-polarization (CP) was applied to all of the samples. The CP contact time was set to 3 ms with a recycle delay of 3 s. Free induction decays (FIDs) were collected over 28,000 scans, resulting in a total acquisition time of ca. 24 h. The spectral width was 30 kHz. Spectra were referenced to tetramethylsilane (TMS) by using adamantane as a secondary reference.

## 3 Results and Discussion

### 3.1 Characterization of the Adsorbent Materials

Figure 2 shows α-chitin, the chitin composite (CC),^45^ and graphene oxide, as well as the synthesized composite material CGO 1:1. As described in our previous work,^45^ CC exhibits an amber coloration, which is likely associated with Maillard-type browning reactions^70–72^ during thermal processing. Under heating conditions, partial deacetylation of GlcNAc units may generate free amino groups capable of reacting with sugar moieties to form conjugated nitrogen-containing chromophores responsible for the observed color. The addition of the GlcNAc monomer is proposed to promote cross-linking between chitin polymer chains, thereby enhancing the mechanical stability of the composite. Thermal treatment may generate reactive dicarbonyl intermediates that facilitate interactions between carbonyl groups of the monomer and partially deacetylated chitin residues promoting Maillard-type reactions.^71–74^ These reactions contribute to network formation.^45^ Similar processes probably happen in the chitin–graphene oxide (CGO) composite. During heating, Maillard-type reactions presumably take place between chitin and GlcNAc, while the oxygen-containing groups of GO can form hydrogen bonds with chitin and GlcNAc. This means that GO can act as an additional interaction point in the network, strengthening the composite as the chitin–GlcNAc chemistry continues.

**Figure 2:**
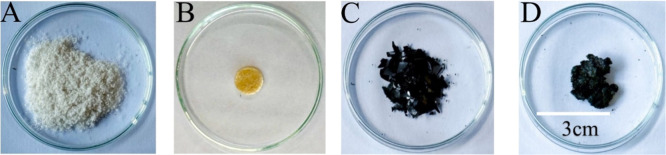
Photographs of (A) α-chitin, (B) chitin composite (CC),^45^ (C) graphene oxide (GO), and (D) CGO 1:1.

The morphological characterization of the chitin/GO (CGO) composites was performed using scanning electron microscopy. α-Chitin forms a network of interlaced disordered fibers aligned in a stacked or twisted arrangement, as observed in Figure 3A,B. In contrast, the SEM images of GO reveal wrinkled or crumpled structures (Figure 3C,D). This wrinkling originates from the interactions between the oxygen-containing functional groups (epoxy, hydroxyl, carboxyl), which disrupt the flat graphene structure. The surfaces of the GO sheets reportedly promote efficient adsorption.^75–77^ In the CGO composite (Figure 3E,F), both morphological features are observed. The composite surface exhibits smooth, sheet-like regions attributable to GO, as well as rougher, fibrous regions corresponding to chitin. The presence of wrinkled lamellar structures intertwined with the fibrous chitin network suggests successful incorporation of GO sheets within the polymer matrix. Unlike GO, no large stacked aggregates are visible, indicating the dispersion of GO throughout the composite. Although distinct phase boundaries are not sharply defined due to nanoscale dispersion, the hybrid morphology combining wrinkled sheets and fibrous structures confirms the formation of a CGO composite.

**Figure 3:**
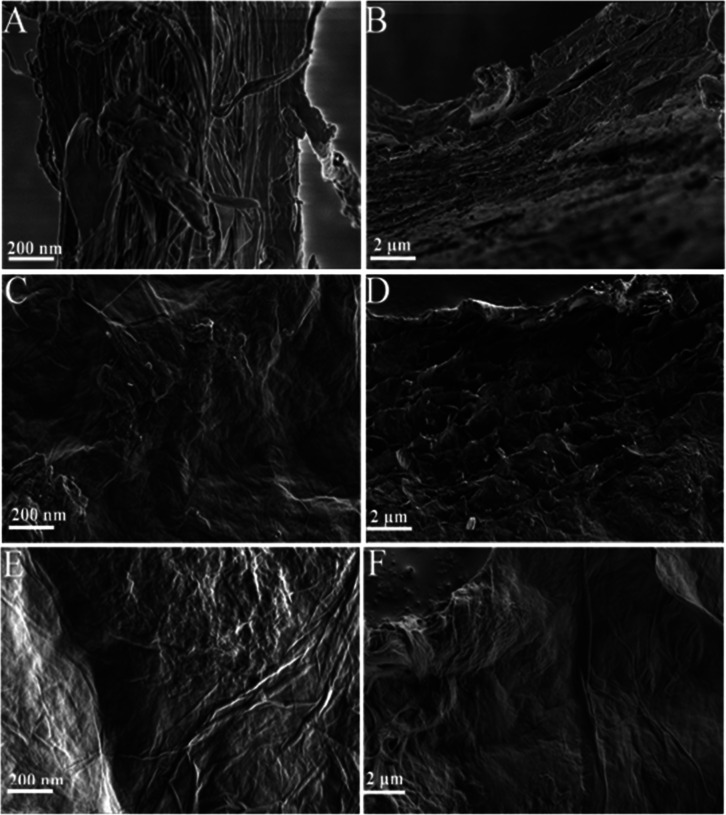
Morphological characterization of α-chitin (A,B), GO (C,D), and CGO 1:1 (E,F) by SEM. Left: (A,C,E) interior and Right: (B,D,F) surface structure of dried α-chitin, GO, and oven-dried CGO 1:1.

To characterize the materials at the molecular level, ^13^C cross-polarization (CP) MAS NMR experiments were performed. Figures 4 and S1 (ESI) show ^13^C CP MAS NMR spectra of α-chitin as well as the CGO composites at different chitin/GO mixing ratios. Spectra exhibit signals from each expected carbon position of α-chitin, indicating that the chitin backbone remains intact, serving as the primary composite constituent, with GO incorporated as the secondary component. Signal assignment is based on the literature^78,79^ (Figure 4 and Table S1 in the Supporting Information).

**Figure 4:**
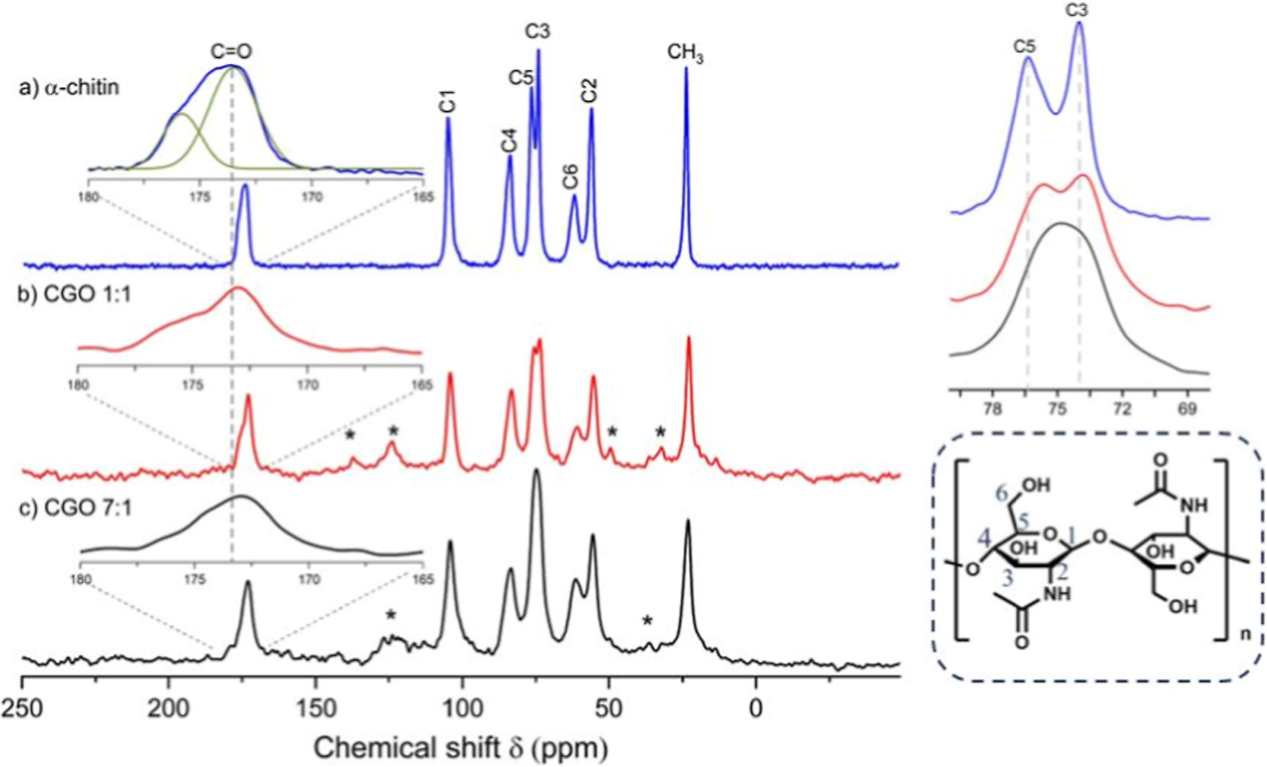
Left: ^13^C CP MAS NMR spectra of (a) α-chitin (this spectrum was adapted from Aravind et al.,^45^ Copyright: published open access by MDPI under Creative Commons CC BY 4.0 license), (b) CGO 1:1, and (c) CGO 7:1. The minor signals indicated by an asterisk in the composite spectrum indicate the spurious residual IL used for processing. Right, top: Magnification of the ^13^C signals C3 + C5. Right, bottom: Structure of α-chitin (adapted from Younes & Rinaudo, 2015,^82^ Copyright: published open access by MDPI under Creative Commons CC BY 4.0 license).

The spectra measured for the α-chitin reference samples agree well with previously published data.^78,80,81^

The main difference between α-chitin and the CGO composites occurs for the carbon signal of C5 and to a lower degree of C3 and C=O (see inserts in Figure 4). The two signals of C3 and C5 merge into one signal due to line broadening and signal shift. There is also a slight difference between α-chitin and the CGO composite for the C=O carbon signal due to the cross-linking of the monomer unit (see insets in Figure 4). The signal shifts to lower chemical shifts and changes shape. However, none of the characteristic signals disappear or shift strongly. This indicates that the molecular structure of chitin remains intact. Only the characteristic hydrogen-bond pattern is potentially influenced, which would explain the observed signal shifts. As expected, no ^13^C signals due to GO could be detected since cross-polarization requires the presence of ^1^H nuclei in the close neighborhood of ^13^C. The carbon environments remain structurally similar across intermediate ratios (2:1 to 5:1), with only differences in signal intensities corresponding to the composition. Consequently, the samples of 1:1 and 7:1 ratio were selected for Figure 4 to represent the compositional extremes: one enriched in graphene oxide and the other dominated by chitin, enabling a clear comparison.

Infrared spectra of CGO samples as well as GO and α-chitin are displayed in Figures 5 and S3 (Supporting Information) with band assignment based on the literature^15,18,83–86^ (see Table S2, Supporting Information). The FTIR spectrum of graphene oxide (GO) displays characteristic absorption bands from oxygen-containing functional groups formed during graphite oxidation. The broad band at about 3400–3300 cm^–1^ is due to O–H stretching.^87^ The peak at 1711 cm^–1^ originates from C=O stretching of groups at the layer edges,^88–90^ which exhibit carboxyl groups. The 1583 cm^–1^ band arises from C=C stretching in aromatic sp^2^ domains of the graphene framework.^91^ The 1344 cm^–1^ band is due to C–OH stretching.^92^ The 1212 cm^–1^ peak arises from the C–O–C stretching of epoxy groups,^47^ and the 1032 cm^–1^ band arises from C–O–C stretching of alkoxy groups.^93^ These bands show that graphite was successfully oxidized to graphene oxide.

**Figure 5:**
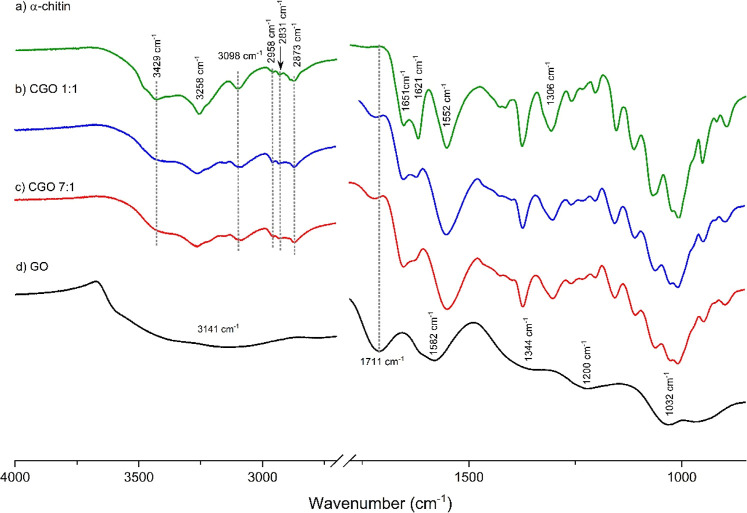
ATR-FTIR spectra of α-chitin (a) (this spectrum was adapted from Aravind et al.,^45^ Copyright: published open access by MDPI under Creative Commons CC BY 4.0 license), CGO 1:1 (b), CGO 7:1 (c), and pure GO (d).

The spectrum of α-chitin displays characteristic absorption bands associated with its polysaccharide backbone and amide functionalities. The bands 3429 and 3259 cm^–1^ are attributed to N–H and O–H stretching vibrations. The 3098 cm^–1^ band is attributed to N–H stretching. Furthermore, aliphatic C–H stretching vibrations appear at 2955–2925 cm^–1^. The two amide bands at 1653 and 1619 cm^–1^ occur due to two different hydrogen bond states.^94^ The component at 1653 cm^–1^ is assigned to C=O groups, hydrogen bonded only to NH groups, while the component at 1619 cm^–1^ is a similar group with another hydrogen bond to side-chain CH_2_OH.^94^ These interchain hydrogen bonds are responsible for the high chemical stability of the α-chitin structure.^15^ The band at 1552 cm^–1^ is assigned to N–H bending coupled with C–N stretching and is called the amide II band. The 1426 cm^–1^ and 1415 cm^–1^ bands originate from CH_2_ bending, and the band at 1375 cm^–1^ is due to CH_3_ bending of the acetamide group. The 1307 cm^–1^ and 1260 cm^–1^ bands correspond to C–N stretching and N–H bending (amide III). The 1234 cm^–1^ band represents C–O stretching in the polysaccharide. The 1154 cm^–1^ peak is from C–O–C stretching of the glycosidic bridge. Bands at 1113 cm^–1^, 1068 cm^–1^, and 1008 cm^–1^ are attributed to C–O stretching of the polysaccharide ring, and the 952 cm^–1^ band is attributed to skeletal vibrations of the carbohydrate backbone. These bands confirm the molecular structure of α-chitin, a polysaccharide with acetamide groups.

The FTIR spectra of CGO composites display bands characteristic of both GO and chitin, thus confirming composite formation. The O–H and N–H stretching band shifts from 3429 cm^–1^ in chitin to 3393–3410 cm^–1^ in CGO, indicating hydrogen bonding between GO and chitin functional groups. The N–H stretching shifts from 3098 cm^–1^ in chitin to approximately 3083–3101 cm^–1^ in CGO, reflecting interactions between chitin N–H groups and GO oxygen-containing groups. The C–H stretching bands typically found between 2900 and 2800 cm^–1^ in α-chitin also exhibit shifts in the CGO composites. The bands in these regions in α-chitin slightly shift to lower values for CGO (1:1) and CGO 7:1. A new peak emerges in the composite around 1710 cm^–1^, which is observed in GO due to C=O stretching of carbonyl groups, but it is not present in α-chitin. This indicates the incorporation of GO in the composites. The C=O band of GO shifts from 1711 cm^–1^ to 1718–1728 cm^–1^ in CGO, suggesting interactions between GO carboxyl groups and the chitin matrix. The characteristic amide I (1650–1653 cm^–1^) and amide II (1550–1552 cm^–1^) bands remain present for CGO, confirming the intact chitin structure.

The presence of the C–O–C band (1154–1165 cm^–1^) and the C–O bands (1100–1000 cm^–1^) indicates that the polysaccharide backbone remains intact. Considering the presence of hydrophilic groups in both components,^95^ chitin supposedly binds to GO through intermolecular hydrogen bonding. Likely interactions could be O–H···O=C, N–H···O=C, and O–H/N–H···O with epoxy and hydroxyl groups of GO. The observed band shifts confirm GO–chitin interactions and successful integration of chitin into the GO matrix. Moreover, major chemical modifications of the constituents can be excluded based on FTIR studies, in agreement with observations from solid-state NMR (see above). Taken together, the observed changes show that the GO is integrated into the chitin, forming a true composite with intermolecular interactions but without any evidence for the formation or breakage of covalent bonds.

### 3.2 Adsorption Studies

Uptake kinetics for Eu­(III) and U­(VI) from solutions was measured as a function of time to evaluate the adsorption behavior of the developed CGO composites (Figure 6).^96^ The initial ion uptake is very rapid and takes place mainly within the first hour, followed by a gradual adsorption over 2 to 5 h (Figure 6A). The system reaches sorption equilibrium after about 12 h. Under the chosen conditions, almost 100% of the metal ions are adsorbed (Figure 6B) for both ions, Eu­(III) and U­(VI). Established pseudo-first-order and pseudo-second-order equations (eqs 3 and 4 above) were tested and compared to fit the experimental data (see also Supporting Information, Figure S5 and Table S3). The correlation coefficient (*R* ^2^) for the pseudo-second-order kinetic model is 0.999, indicating an almost perfect agreement between the experimental adsorption data and the model for Eu­(III) and U­(VI). The pseudo-first-order kinetic model shows a somewhat lower agreement, i.e., *R* ^2^ values of 0.985 for Eu­(III) and 0.956 for U­(VI) (see the Supporting Information). It should be noted that these models are well-established for describing ion adsorption processes from solutions.^97^ It is likely that the adsorption mechanism is primarily driven by physisorption since the CGO composites contain various interaction sites that may be charged or uncharged depending on pH (cf. below). Metal ions can thus be adsorbed through electrostatic attraction and other surface interaction mechanisms such as hydrogen bonding, as reported for graphene-based adsorbents.^98^ Kinetics studies took place at pH 6 for uranium and pH 5 for europium. Uranium mainly occurs as uranyl species (UO_2_^2+^ and UO_2_OH^+^) at a pH of 6. Europium mainly forms Eu^3+^ at a pH of 5.

**Figure 6:**
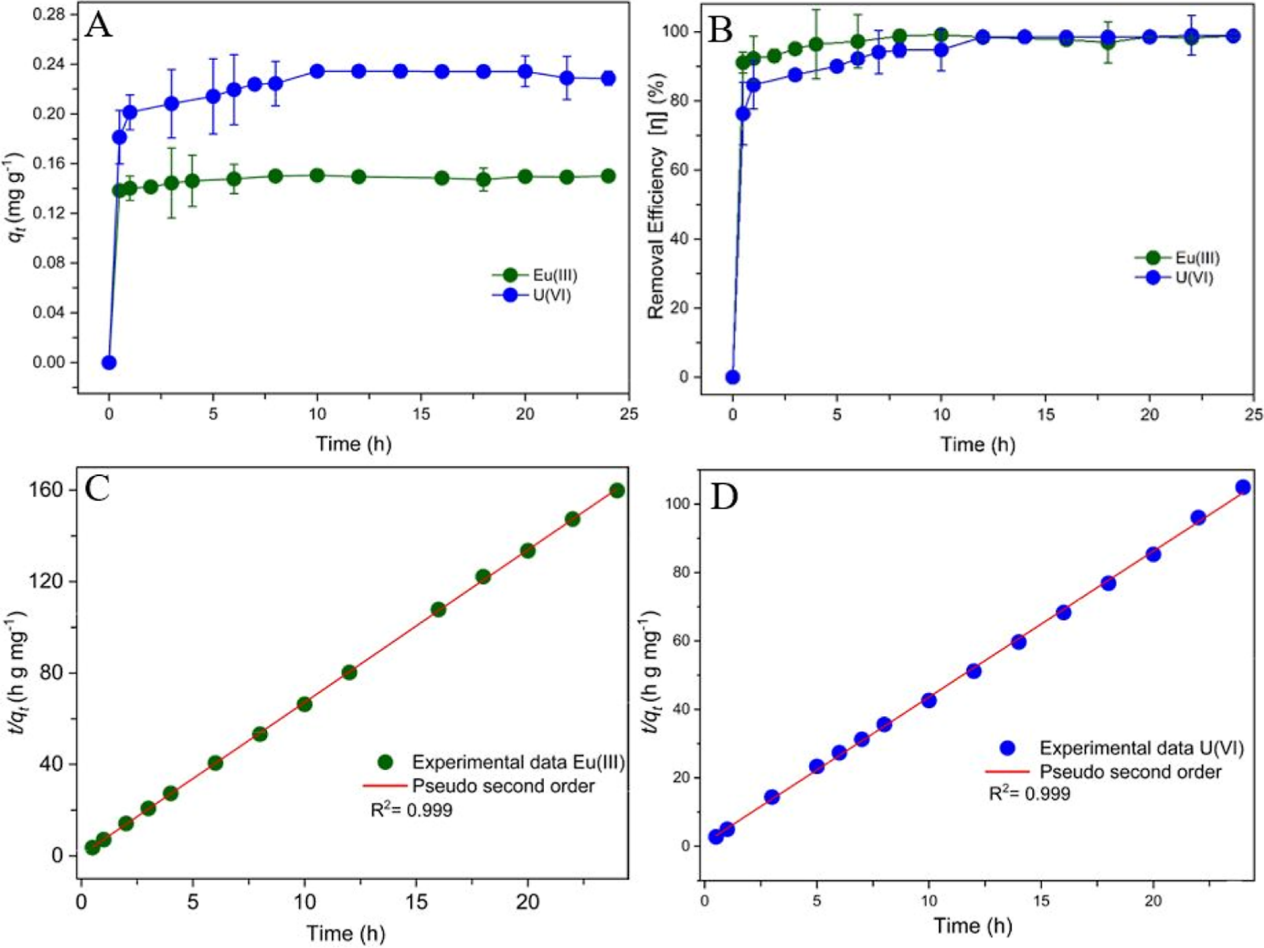
Adsorption kinetics for CGO 7:1. The removal of Eu­(III) and U­(VI) by the composite is demonstrated. (A) Uptake *q*_t_ in mg g^–1^. (B) Removal efficiency R % in percent of the initial ion concentration in solution. (C,D) Fitted data using the pseudo-second-order model (cf. eq 4).

The positive influence of GO incorporation on the adsorption behavior is demonstrated further by comparing different adsorbents after 1 and 7 day Eu­(III) adsorption (Figure 7). A direct comparison between GO and the CGO composite indicates that both achieve similarly high removal efficiencies, with nearly complete adsorption occurring within 1 day. The CGO composites exhibit an adsorption performance comparable to that of GO, indicating that GO incorporation into the chitin matrix enhances the adsorption capacity while preserving GO’s high efficiency. Notably, both CGO composites adsorb significantly larger amounts in much shorter times than pure chitin and especially than the previously synthesized chitin-based composite (CC).^45^ This indicates that GO with its oxygen-containing groups strongly enhances the adsorption capacity. Even for the incorporation of only 12.5% GO, i.e., for a chitin/GO mixing ratio of 7:1, practically complete removal of Eu­(III) is observed (see also the kinetics experiments, Figure 6).

**Figure 7:**
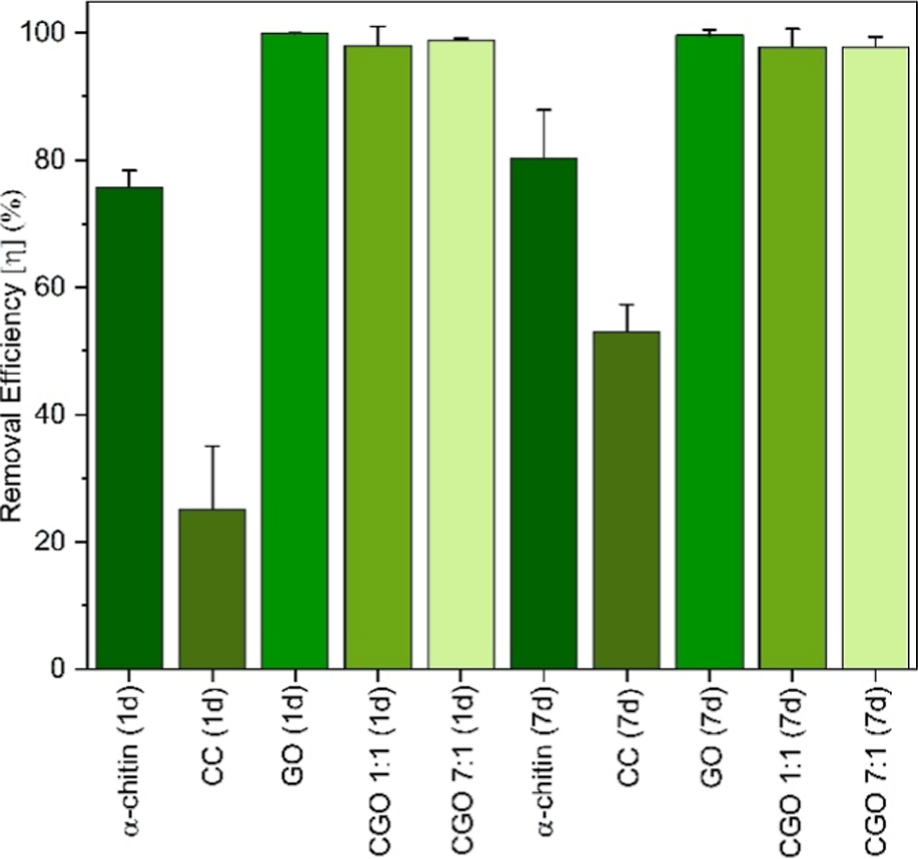
Eu­(III) removal efficiency by chitin, purely chitin-based composites (CC), data taken from ref 45, pure GO, and CGO 1:1 and 7:1 after 24 h and 7 d for *C*_0_ (EuCl_3_.6 H_2_O) = 10^–5^ M.

Similar results are obtained for U­(VI) (see Figure 8), and the CGO composite adsorbs even slightly more uranium than pure GO after 7 days.

**Figure 8:**
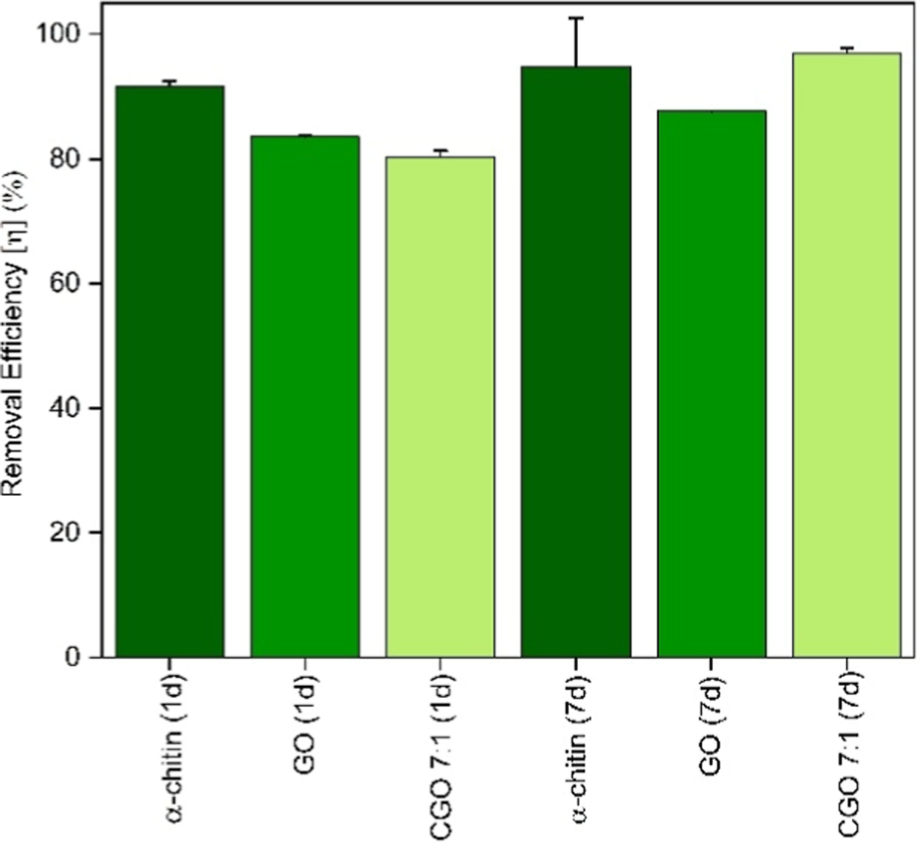
U­(VI) removal efficiency by chitin, GO, and CGO 7:1 after 24 h and 7 d for *C*_0_ (UO_2_(NO_3_)_2_) = 10^–5^ M.

Further studies were performed to evaluate the influence of adsorbent dosage, solution pH, and initial metal concentration on metal-ion removal using Eu­(III) (see Figure 9). To study the influence of adsorbent dosage and avoid adsorbent-excess conditions, these additional experiments were conducted using 10 mL of solution, varying the CGC amount between 1 and 15 mg. As shown in Figure 9A, the removal efficiency linearly increases with the adsorbent mass, increasing from approximately 37% for 1 mg to about 85% for 15 mg.

**Figure 9:**
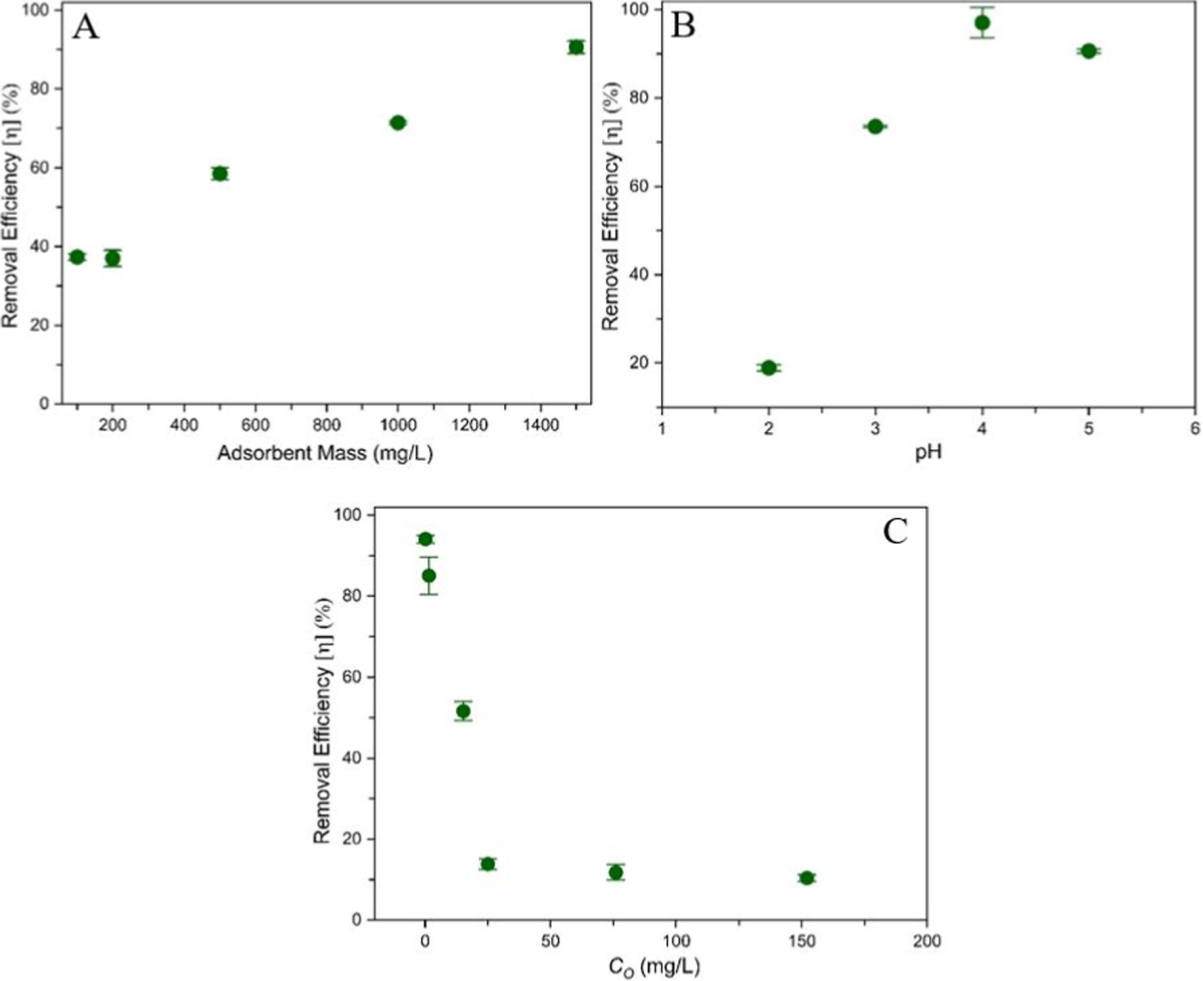
Eu­(III) removal efficiency of CGO 7:1. (A) Effect of CGO mass (pH 5.1, 25 °C, *V* = 10 mL, contact time: 1 day); (B) effect of pH (25 °C, CGO mass: 15 mg, *V* = 10 mL, contact time: 1 day); and (C) effect of initial Eu­(III) concentration *C*_O_ (pH 5.1, 25 °C, CGO mass: 15 mg, contact time: 1 day, *V* = 10 mL).

Metal-ion adsorption on CGO composites is strongly pH-dependent (cf. Figure 9B). The removal efficiency increases significantly from about 18% at pH 2 to about 97% at pH 4, followed by a slight decrease at pH 5. At pH 2, H^+^ ions compete with Eu­(III) ions for available adsorption sites on the adsorbent surface. Functional groups such as COOH and OH remain protonated at low pH, leading to a decrease in negative surface charge. This significantly reduces the adsorption capacity. As the pH increases, these groups start to deprotonate, thus enhancing electrostatic attraction and promoting the binding of metal ions.^99^ This is particularly true for COOH groups of GO, which provide negatively charged interaction sites for metal ions. Moreover, the interaction with uncharged functional groups occurs through weak coordinative interactions. At higher pH, europium hydroxide species start to form, which reduces the number of free europium ions and finally causes europium hydroxide precipitation. Experiments beyond pH 5 were thus not conducted.^100^

In summary, the CGO adsorbents are shown to be highly efficient under acidic conditions, i.e., between pH 3 and 5. The observed pH dependence is mainly due to changes of effective surface charges, and the observed behavior aligns well with previous studies on related materials.^101–109^

The initial Eu­(III) concentration *C*_O_ was varied between 0.15 and 150 mg L^–1^ (see Figure 9C). The removal efficiency for 15 mg CGO 7:1 exceeds 90% for low *C*_O_ values. It then drops down to about 10% at *C*_O_ ≥ 25 mg L^–1^. Obviously, the adsorption sites are increasingly saturated at higher *C*_O_ values, thus reducing the overall removal efficiency. This means that the CGO 7:1 material is well-suited to remove spurious amounts of Eu­(III) very efficiently from aqueous solutions.

The equilibrium adsorption isotherm was determined by variation of the initial Eu­(III) concentration. Data were analyzed using the Langmuir isotherm (eq 5), as shown in Figure 10 and Table S4 (Supporting Information). This model assumes monolayer adsorption on a homogeneous surface with identical adsorption sites.^110–113^ For comparison, the data were also analyzed using the Freundlich isotherm (see Supporting Information, Figure S6 and Table S4). The correlation coefficient for the Langmuir model (*R* ^2^ = 0.966) is higher than for the Freundlich model (*R* ^2^ = 0.903), indicating that the Langmuir model better describes the adsorption isotherm. This suggests that adsorption occurs on homogeneous adsorbent surfaces predominantly as monolayer coverage, i.e., no further adsorption occurs once an adsorption site is occupied. As discussed above, the composite contains various functional groups where adsorbate molecules can individually bind via electrostatic interactions and other interactions like hydrogen bonding. Once occupied, these sites cannot adsorb additional molecules, consistent with the Langmuir monolayer adsorption model.

**Figure 10:**
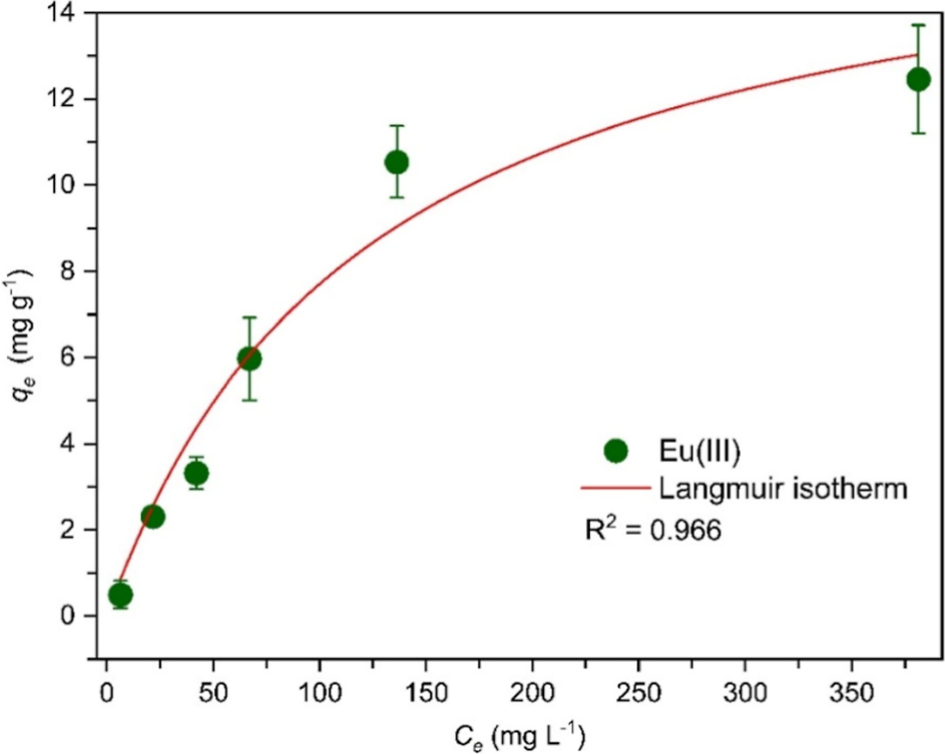
Eu­(III) adsorption isotherm on CGO 7:1 and Langmuir isotherm fit (25 °C, pH 5.1, CGO mass: 15 mg, contact time: 1 day, *V* = 10 mL).

The Freundlich model also provides interesting information: The determined Freundlich constant *n* amounts to 1.97. Its inverse value is indicative of favorable (0.1 < 1/*n* < 0.5) and unfavorable (1/*n* > 2) adsorption processes. The observed value of 1/*n* = 0.51 thus indicates rather favorable adsorption, i.e., a relatively strong affinity between Eu­(III) and the CGO composite surface.^114–116^

The maximum Eu­(III) adsorption capacity *q*_m_ of 17.2 mg g^–1^ determined for CGO 7:1 from the Langmuir model (see Supporting Information, Table S4) is comparable with that of other reported adsorbents (see Table 1), such as GO@CS composite beads (23.2 mg g^–1^), the cellulose–yeast biosorbent (25.9 mg g^–1^), and thiourea-functionalized cellulose (27 mg g^–1^). Other materials listed in Table 1 exhibit even higher adsorption capacities.

**Table 1:** Comparison of Different Reported Adsorbents^a^

Adsorbents	Ion	*q*_*m*_ (mg g^–1^)	pH	Time	Environmental Aspect	References
GO–maghemite	Eu(III)	54 −103	6–7	30–60 min	Biopolymer composite	Lujanienė et al.^105^
Cellulose–yeast	Eu(III)	25.9	5–6	∼120 min	Renewable biomass	Arunraj et al.^117^
GO@CS composite beads	Methylene blue	23.3	7–8	∼60 min	Biopolymer composite	Nayl et al.^118^
Tin molybdate talc	Eu(III)	28.0	5–6	60–90 min	Natural mineral	Abass et al.^119^
SBA15-NH-PMIDA	Eu(III)	86	4–5	∼60 min	Mesoporous silica	Fonseka et al.^120^
HCM	Eu(III)	176.3	5–6	∼60 min	Designed hybrid adsorbent	Awual et al.^121^
Arthrospira platensis	Eu(III)	9.8–29.8	5–6	60–120min	Algal biosorption	Yushin et al.^122^
Cellulose–Saccharomyces cerevisiae	Eu(III)	27	∼5	∼120 min	Biodegradable polymer	Negrea et al.^123^
CGO composite	Eu(III)	17.2	3–5	30–60 min	Biopolymer composite	This work

a Maximum adsorption capacities (*q*_*m*_), optimal pH ranges, contact times, and environmental aspects are provided.

It should, however, be noted that the material studied here contains only a relatively low amount of GO (chitin/GO ratio = 7:1). Higher GO contents are likely to result in higher adsorption capacities. Furthermore, the CGO composite stands out with respect to its pH range of 3–5 for efficient ion removal and the short contact time required for Eu­(III) removal from aqueous solutions.

### 3.3 Characterization of the Adsorbent after Adsorption and Reuse

To ensure the structural integrity of the adsorbents after europium and uranium sorption, we also measured ^13^C CP MAS NMR and ATR-IR spectra (see Supporting Information, Figures S2 and S4) to allow for comparison with those of the unloaded samples discussed above (Figures 4 and 5). Adsorption of europium and uranium did not result in significant changes of the NMR spectra, apart from decent line broadening, caused by the paramagnetic nature of Eu (III) and U­(VI) species. This observation suggests that these metals are not covalently bound to chitin as the formation of chemical bonds would typically produce measurable chemical shifts or signal splitting in the NMR spectra. Similarly, ATR-FTIR spectra did not show substantial structural changes after adsorption. The notable exception was a band around 1710 cm^–1^ in the composite, attributed to C=O stretching of protonated carboxylic acid groups in GO. This band disappeared after adsorption, indicating the involvement of carboxyl-containing functional groups in the binding process. This observation suggests the direct participation of GO carboxyl-containing functional groups in metal binding and indicates that adsorption performance is influenced by surface-accessible oxygen-containing functional groups, likely via coordination or electrostatic interactions with metal species (see above). Notably, the Eu­(III)- and U­(VI)-loaded samples exhibit shifts of the OH/NH, amide, and C–O-related bands in the FTIR spectra, confirming the participation of these groups in metal adsorption. For Eu­(III), the broad OH/NH band shifts from 3369 cm^–1^ to 3431 cm^–1^ after 1 day, indicating ion interaction with hydroxyl groups. The absence of other significant spectral changes confirms that ion adsorption does not cause chemical modification or degradation of the chitin backbone. Overall, these observations confirm that sorption is mainly governed by physical interactions rather than strong chemical bonding. Such weak interactions tend to favor the reversible adsorption/desorption of the metal. The adsorption–desorption performance of CGO 7:1 for europium was assessed over two consecutive cycles using nitric acid (HNO_3_) solutions of two different concentrations, namely, 0.05 and 0.2 M (Figure 11).

**Figure 11:**
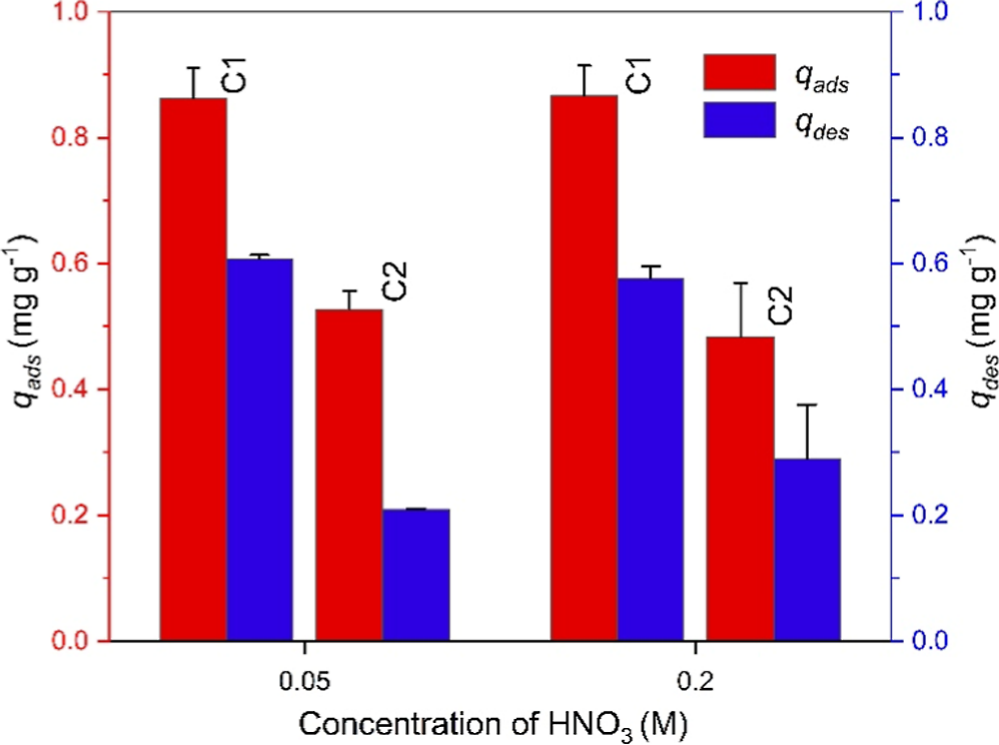
Adsorption (*q*_*ads*_) and desorption (*q*_*des*_) capacities of CGO 7:1 for Eu­(III) over two cycles using different HNO_3_ concentrations for desorption.

Desorption of Eu­(III) from CGO then supposedly occurs through protonation of negatively charged surface functional groups under acidic conditions. Their protonation thus weakens electrostatic interactions, facilitating the release of Eu­(III) ions into solution. Treatment with HNO_3_ after the first adsorption cycle resulted in the release of about 3/4 of the adsorbed Eu­(III), i.e., about 1 remained adsorbed for both HNO_3_ concentrations. The adsorption capacity observed in cycle 2 was then approximately the same value as that removed by HNO_3_ treatment. It can thus be stated that the overall Eu­(III) loading was similar to the amount observed after cycle 1.

However, it should be noted that the acid treatment also caused a loss of the adsorbent material. The composite mass decreased from 15 mg in cycle 1 to ca. 10 mg after regeneration with 0.05 M HNO_3_ and 5 mg after regeneration with 0.2 M HNO_3_. This indicates that higher HNO_3_ concentrations lead to partial degradation of the CGO composite. The observed mass loss is likely attributed to partial hydrolysis of chitin, which can lead to degradation of the chitin matrix and weakening of the interactions between chitin and graphene oxide,^54,124^ especially at the higher HNO_3_ concentration. Therefore, 0.05 M HNO_3_ appears to be better at preserving the material.

## 4 Conclusions

The present study reports a novel composite material comprising commercially available α-chitin and graphene oxide, prepared as stable materials via dissolution in IL [BMIM]­[OAc]. The obtained CGO composite materials possess stable structures and are suitable for applications such as adsorption-based filter media. The structure of the synthesized adsorbent was verified using FTIR, SEM, and solid-state NMR analyses. Incorporation of GO into the chitin matrix enhances the adsorption performance of the composite materials. Notably, even a relatively small fraction of GO substantially increases the adsorption efficiency compared to pure chitin composites (CC). Given the high cost of GO, these results are promising as the composites maintain high effectiveness even when composed largely of the cost-effective and sustainable chitin component. The adsorption performance of the CGO composites was assessed for the removal of Eu­(III) and U­(VI) ions from aqueous solutions. The results indicate that the material is highly efficient, especially in eliminating low concentrations of these metal ions. The adsorption process follows the pseudo-second-order kinetic model, confirming that physisorption is the predominant mechanism. Almost complete uptake is reached within only 30–60 min contact time. The adsorption isotherm is well-described by the Langmuir model, yielding a theoretical maximum Eu­(III) adsorption capacity of 17.2 mg g^–1^ for CGO 7:1. Moreover, the CGO adsorbent efficiently works in the pH range of 3–5, and the CGO composite can be readily separated from water after use and retains its structural integrity.

Regeneration studies demonstrated that effective desorption can be achieved at lower HNO_3_ concentrations, emphasizing the importance of optimizing regeneration conditions to maintain the performance over repeated cycles.

In summary, novel CGO composites are prepared in ionic liquids under mild reaction conditions, mainly from the readily available biopolymer chitin by adding relatively low amounts of graphene oxide. The obtained materials exhibit fast uptake kinetics, effective ion removal properties even at relatively low pH, down to 3, and a moderate maximum adsorption capacity. These properties render the CGO composites particularly promising for the removal of low heavy-metal-ion concentrations from less contaminated waters. In the case of heavily contaminated wastewaters, CGO composites may be used for a highly efficient second cleaning step after the removal of major contaminants in a first step.

## Supplementary Material



## Data Availability

All data supporting the findings of this study are available within the main manuscript and the Supporting Information.

## Notes

^13^C CP/MAS NMR spectra of CGO composites with different chitin:GO mixing ratios, along with ^13^C CP/MAS NMR spectra of the adsorbent before and after adsorption, and a table of ^13^C signal assignments for α-chitin and CGO composites; ATR-FTIR spectra of CGO composites at different chitin:GO mixing ratios and ATR-FTIR spectra of the adsorbent before and after Eu­(III) adsorption, including a table of IR band assignments based on the literature; and adsorption studies, including experimental data and analysis (PDF)
