# Supplementary material for: Chitin/Graphene Oxide Composite Materials for Heavy-Metal-Ion Adsorption
Source: ACS Omega. 2026 May 21;11(21):31182–96. doi: 10.1021/acsomega.6c00814 (PMC13234898; doi:10.1021/acsomega.6c00814)
Supplement: Supplementary file 1 [file ao6c00814_si_001.pdf]

Electronic Supporting Information

for

# Chitin/Graphene Oxide Composite Materials for Heavy Metal Ion Adsorption

*Anjana Aravind,*[1] *Kevin Gerein,*[2] *Andreas Hirsch,*[2] *Frank Hauke,*[2] *and Eike Brunner*[1*]

[1] Chair of Bioanalytical Chemistry, Faculty of Chemistry and Food Chemistry, TU Dresden, 01062 Dresden, Germany

[2] Chair of Organic Chemistry II, Department of Chemistry and Pharmacy, FAU Erlangen-Nürnberg, 91058 Erlangen, Germany

# Table of Contents

1. $^{13}C$ CP/MAS NMR spectra of CGOs with different chitin:GO mixing ratios............. S03
2. $^{13}C$ CP/MAS NMR spectra of the adsorbent before and after adsorption .................... S04
3. Assignment of $^{13}C$ CP/MAS NMR signals of α-chitin and CGO composites............. S05
4. ATR-FTIR spectra of CGOs with different chitin:GO mixing ratios ........................... S06
5. ATR-FTIR spectra of the adsorbent before and after adsorption................................. S07
6. IR band assignments based on literature ........................................................................ S08
7. Adsorption Kinetics ...................................................................................................... S09
8. Adsorption Isotherm ..................................................................................................... S011
9. References..................................................................................................................... S013

## 1. $^{13}$C CP MAS NMR) spectra of CGOs with different chitin:GO mixing ratios

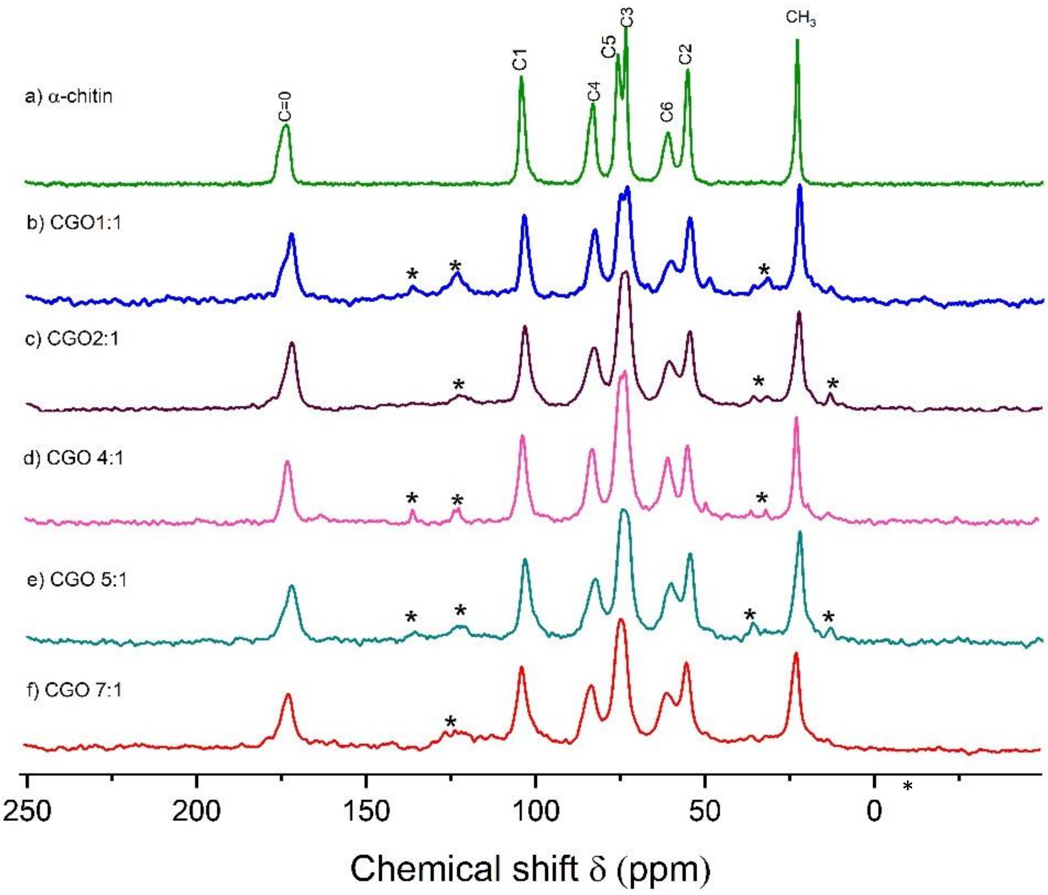


**Figure S1:** $^{13}$C CP MAS NMR spectra (a) pure α-chitin, (b) chitin/GO composite with mixing ratio 1:1 (CGO 1:1), (c) CGO 2:1, (d) CGO 4:1, (e) CGO 5:1, (f) CGO 7:1. The minor signals indicated by an asterisk in the composite spectrum are due to residual spurious amounts of the ionic liquid used for processing.

## 2. $^{13}C$ CP MAS NMR spectra of the adsorbent before and after adsorption

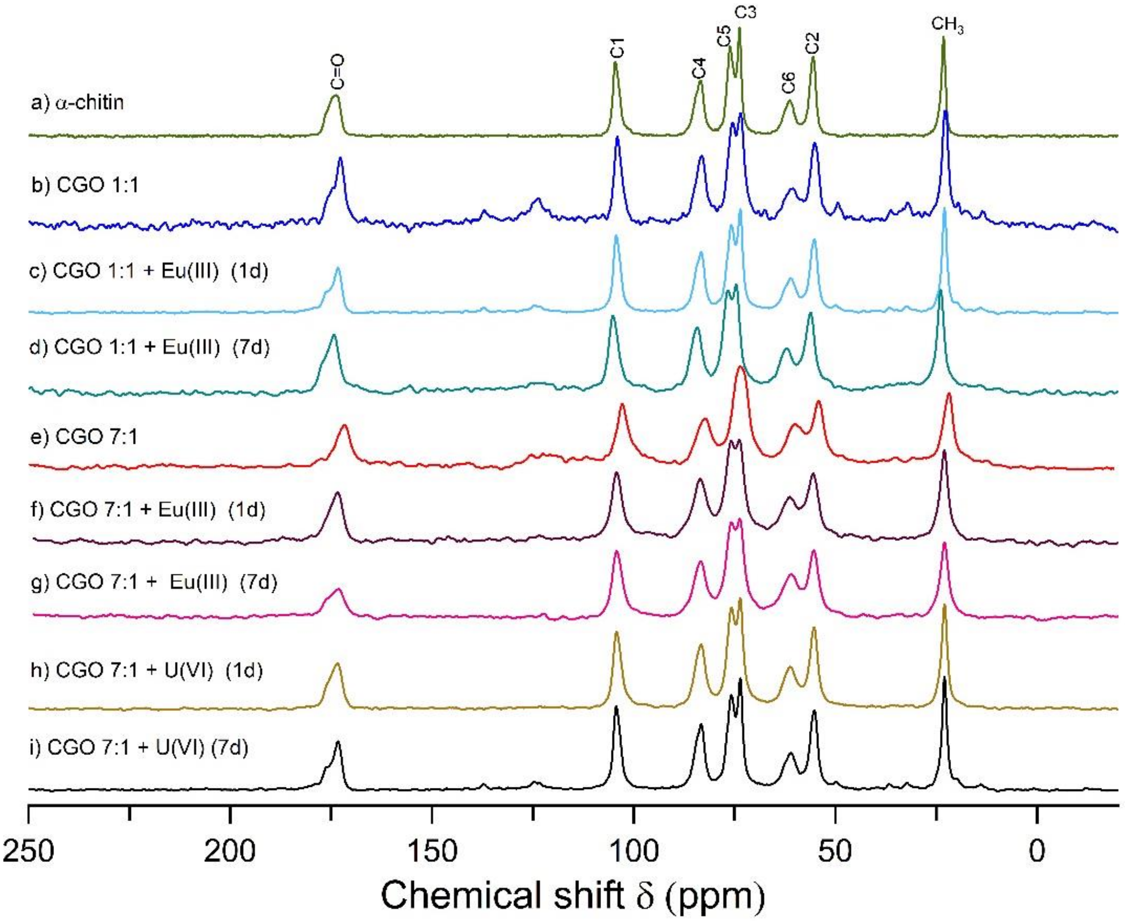


**Figure S2:** $^{13}C$ CP MAS NMR spectra of (a) pure α-chitin, (b) CGO 1:1, (c) after Eu(III) adsorption for 1d, (d) after 7d Eu(III) adsorption; (e) CGO 7:1, (f) after Eu(III) adsorption for 1d, (g) after 7d Eu(III) adsorption, (h) CGO 7:1 after U(VI) adsorption for 1d, (i) CGO 7:1 after 7d U(VI) adsorption.

# 3. [13]C signal assignment of $\alpha$-chitin and CGO composites before and after adsorption

The $^{13}$C signal assignment of $\alpha$-chitin and CGO composites from the spectra are summarized and given below:[1]

| Sample | C=O | | C-1 | | C-4 | | C-5 | | C-3 | | C-6 | | C-2 | | $CH_3$ | |
|---|---|---|---|---|---|---|---|---|---|---|---|---|---|---|---|---|
| | δ (ppm) | $\Delta\nu_{1/2}$ (Hz) | δ (ppm) | $\Delta\nu_{1/2}$ (Hz) | δ (ppm) | $\Delta\nu_{1/2}$ (Hz) | δ (ppm) | $\Delta\nu_{1/2}$ (Hz) | δ (ppm) | $\Delta\nu_{1/2}$ (Hz) | δ (ppm) | $\Delta\nu_{1/2}$ (Hz) | δ (ppm) | $\Delta\nu_{1/2}$ (Hz) | δ (ppm) | $\Delta\nu_{1/2}$ (Hz) |
| **α-chitin** | 174 | 264.4 | 104.2 | 196.5 | 83.5 | 241.1 | 75.7 | 244.1 | 73.7 | 205.6 | 61.2 | 384 | 55.4 | 233 | 23 | 162.2 |
| **CGO (1:1)** | 173 | 266.8 | 104 | 189.4 | 83.3 | 268.9 | 75.3 | 258.9 | 73.7 | 233 | 60.5 | 446.4 | 55.2 | 251.3 | 23.1 | 189.4 |
| **CGO (2:1)** | 172.3 | 225.2 | 104 | 231.4 | 83 | 370.4 | 75.1 | 275.5 | 73.5 | 234.5 | 61 | 415.4 | 55 | 253.3 | 22.6 | 209.6 |
| **CGO (4:1)** | 173.5 | 186.7 | 104 | 185 | 83.1 | 247.4 | 74.8 | 250.7 | 73.2 | 238.7 | 60.4 | 329 | 55 | 232.9 | 22.8 | 135 |
| **CGO (5:1)** | 172.7 | 281.2 | 104.2 | 237.2 | 83 | 378 | 75.5 | 278.1 | - | - | 61 | 440.5 | 55.3 | 246.6 | 22.8 | 214 |
| **CGO (7:1)** | 173.2 | 287.95 | 104 | 310.2 | 83.6 | 364 | 74.8 | 326.3 | - | - | 60.8 | 484.5 | 55.6 | 304.1 | 22.9 | 234.7 |
| **CGO (1:1) Eu(III) (1d)** | 173.5 | 203.3 | 104 | 189.9 | 83.5 | 279.8 | 75.5 | 251.7 | 73.8 | 197.32 | 60.7 | 364.6 | 55.4 | 193.2 | 23.3 | 155.8 |
| **CGO (1:1) Eu(III) (7d)** | 173.6 | 264.4 | 104 | 196.5 | 83.3 | 241.1 | 75.5 | 244.2 | 73.8 | 205.6 | 60.9 | 384 | 55.3 | 233 | 23 | 162.2 |
| **CGO (7:1) Eu(III) (1d)** | 173.3 | 251.7 | 104.2 | 232.4 | 83.5 | 342.3 | 75.8 | 306 | 73.7 | 265.4 | 61.3 | 503.3 | 55.5 | 286.4 | 23 | 175 |
| **CGO (7:1) Eu(III) (7d)** | 173.1 | 297.2 | 104.2 | 207.2 | 83.3 | 302.3 | 75.8 | 282.8 | 73.3 | 243.6 | 61 | 477.1 | 55.4 | 252 | 23 | 179.5 |
| **CGO (7:1) U(VI) (1d)** | 173.4 | 288.6 | 104.2 | 165.9 | 83.3 | 238.9 | 75.8 | 175.8 | 73.6 | 100.8 | 61.2 | 338.2 | 55.3 | 112.6 | 23 | 153.1 |
| **CGO (7:1) U(VI) (7d)** | 173.3 | 288.6 | 104.3 | 165.9 | 83.3 | 238.9 | 75.8 | 175.8 | 73.6 | 100.8 | 61 | 338.2 | 55.3 | 112.6 | 23 | 153.1 |

**Table S1:** Chemical shifts and line widths of the $^{13}$C NMR signal observed for the pure α-chitin, chitin/GO-based composite of different mixing ratios: 1:1, 2:1, 4:1, 5:1, 7:1, after Eu(III) adsorption for 1d, after 7d Eu(III) adsorption for CGO 1:1 and 7:1, adsorption for 1d, after 7d U(VI) adsorption for CGO 7:1.

## 4. ATR-FTIR spectra of CGO with different chitin:GO mixing ratios

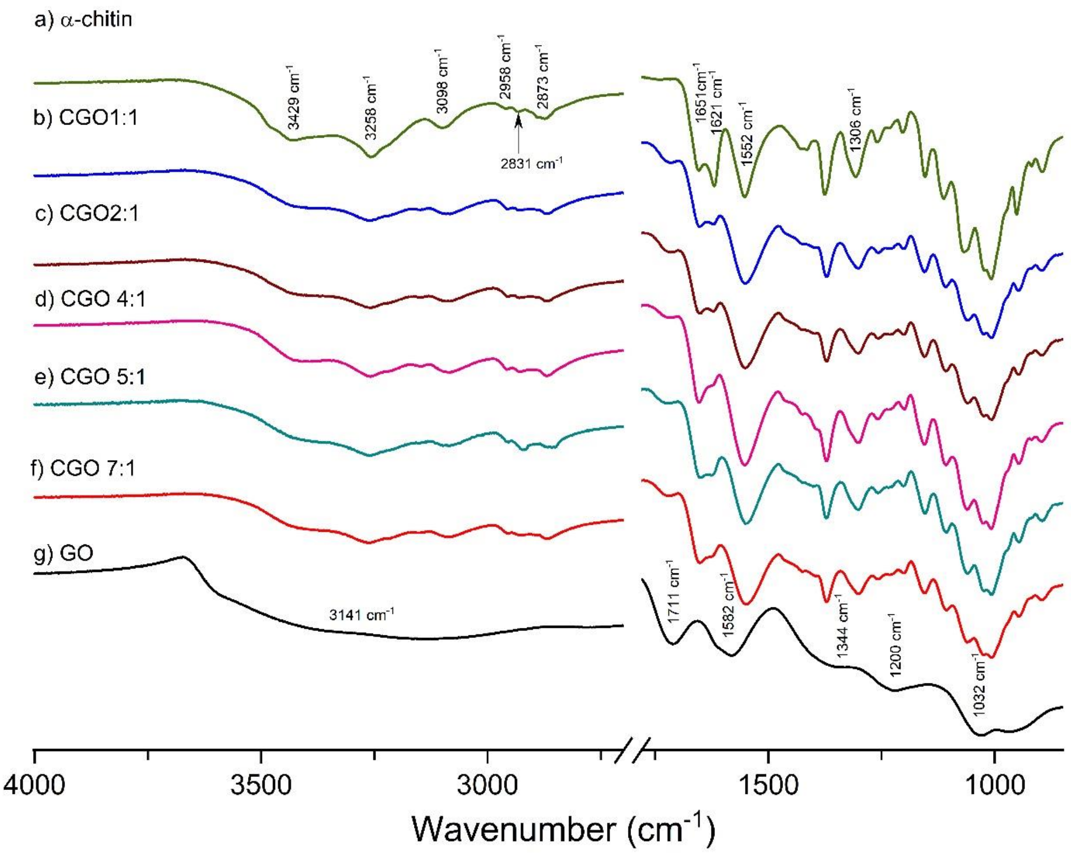


**Figure S3:** ATR-FTIR spectrum of (a) pure α-chitin, (b) CGO 1:1, (c) CGO 2:1, (d) CGO 4:1, (e) CGO 5:1, (f) CGO 7:1, (g) GO.

## 5. ATR-FTIR spectra of the adsorbent before and after Eu (III) adsorption

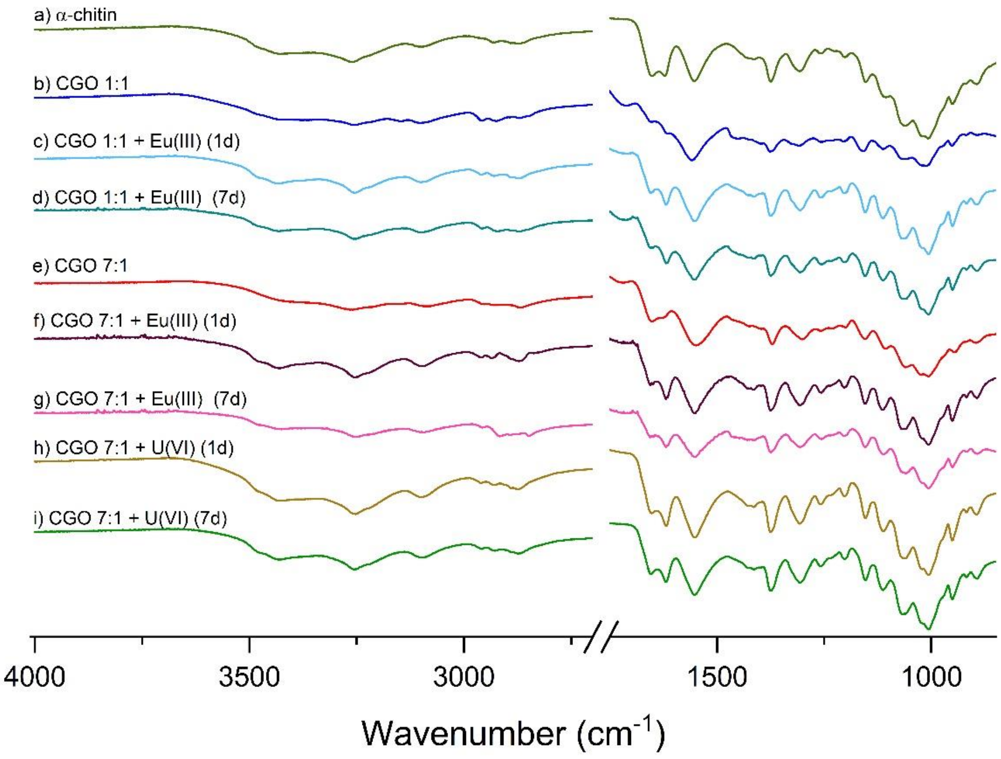


**Figure S4:** ATR-FTIR spectrum of (a) pure α-chitin, (b) CGO 1:1, (c) after Eu (III) adsorption for 1d, (d) after 7d Eu (III) adsorption; (e) CGO 7:1, (f) after Eu(III) adsorption for 1d, (g) after 7d Eu(III) adsorption, (h) CGO 7:1, after U(VI) adsorption for 1d, (i) CGO 7:1 after 7d U(VI) adsorption.

## 6. IR band assignment composites before and after adsorption based on the literature:

The IR band assignment of *α*-chitin and CGO composites before and after adsorption from the spectra are summarized and given below:[2–8]

| Assignment | GO ($cm^{-1}$) | α-chitin ($cm^{-1}$) | CGO 1:1 ($cm^{-1}$) | CGO 2:1 ($cm^{-1}$) | CGO 4:1 ($cm^{-1}$) | CGO 5:1 ($cm^{-1}$) | CGO 7:1 ($cm^{-1}$) | CGO 1:1 Eu(III) (1d) ($cm^{-1}$) | CGO 1:1 Eu(III) (7d) ($cm^{-1}$) | CGO 7:1 Eu(III) (1d) ($cm^{-1}$) | CGO 7:1 Eu(III) (7d) ($cm^{-1}$) | CGO 7:1 U(VI) (1d) ($cm^{-1}$) | CGO 7:1 U(VI) (7d) ($cm^{-1}$) |
|---|---|---|---|---|---|---|---|---|---|---|---|---|---|
| $\upsilon_{OH}$ | 3400 | 3429 | 3393 | 3409 | 3410 | 3409 | 3369 | 3430 | 3385 | 3431 | 3425 | 3423 | 3431 |
| $^{as}\upsilon_{NH}$ | 3300 | 3259 | 3256 | 3259 | 3259 | 3259 | 3259 | 3255 | 3256 | 3254 | 3256 | 3252 | 3256 |
| $^{s}\upsilon_{NH}$ | | | 3148 | 3150 | 3153 | 3150 | | | | | | | |
| $^{s}\upsilon_{NH}$ | | 3098 | 3083 | 3080 | 3098 | 3086 | 3094 | 3101 | 3100 | 3097 | 3101 | 3100 | 3095 |
| $^{as}\upsilon_{CH3}$ | | 2959 | 2957 | 2958 | 2954 | 2954 | 2953 | 2960 | 2957 | 2962 | 2957 | 2960 | 2959 |
| $^{s}\upsilon_{CH2}$ | | 2931 | 2930 | 2930 | 2922 | 2922 | 2926 | 2930 | 2923 | 2933 | 2916 | 2931 | 2927 |
| $^{s}\upsilon_{CH3}$ | | 2873 | 2870 | 2869 | 2867 | 2868 | 2869 | 2873 | 2869 | 2871 | 2849 | 2874 | 2874 |
| C=O | 1711 | | 1720 | 1718 | 1728 | 1713 | 1720 | 1763 | 1762 | | | | |
| $\upsilon_{C=O}$ (Amide I) | | 1653 | 1652 | 1653 | 1653 | 1650 | 1650 | 1655 | 1655 | 1642 | 1655 | 1652 | 1656 |
| $\upsilon_{C=O}$ (Amide I) | | 1619 | 1622 | 1621 | | 1625 | 1628 | | 1618 | | | 1618 | 1618 |
| $\nu_{C=C}$ | 1583 | | | | | | | | | | | | |
| $\delta_{NH} + \nu_{CN}$ (Amide II) | | 1552 | 1551 | 1552 | 1550 | 1552 | 1549 | 1550 | 1550 | 1550 | 1550 | 1552 | 1552 |
| $\delta_{CH2}$ | | 1426 | 1425 | 1424 | | 1424 | 1423 | 1426 | | | | 1426 | 1426 |
| | | 1415 | 1397 | | 1401 | | | 1413 | 1414 | 1415 | 1412 | 1414 | 1414 |
| $\upsilon_{CO}$ | 1344 | | | | | | | | | | | | |
| $\delta_{CH} + \delta_{C\text{-}CH3}$ | | 1375 | 1371 | 1371 | 1371 | 1371 | 1371 | 1375 | 1375 | 1371 | 1374 | 1375 | 1375 |
| $\nu_{CN} + \delta_{NH}$ amide III | | 1307 | 1302 | 1301 | 1302 | 1301 | 1300 | 1307 | 1306 | 1307 | 1307 | 1306 | 1307 |
| $\delta_{NH}$ | | 1260 | 1257 | 1248 | 1258 | 1258 | 1258 | 1258 | 1258 | 1256 | 1258 | 1259 | 1257 |
| $\upsilon_{(COC)}$ | 1212 | | | | | | | | | | | | |
| $^{as}\upsilon_{COC}$ | | 1154 | 1155 | 1155 | 1155 | 1165 | 1155 | 1154 | 1155 | 1154 | 1154 | 1154 | 1153 |
| $\upsilon_{CO}$ | | 1113 | 1108 | 1108 | 1107 | 1108 | 1107 | 1113 | 1113 | 1113 | 1112 | 1112 | 1113 |
| | | 1068 | 1068 | 1061 | 1069 | 1068 | 1069 | 1068 | 1060 | 1068 | 1059 | 1060 | 1067 |
| | | 1018 | 1025 | 1025 | 1025 | 1033 | 1024 | 1019 | 1024 | 1022 | 1024 | 1019 | |
| | | 1008 | 1008 | 1008 | 1008 | 1008 | 1007 | 1006 | 1006 | 1008 | 1008 | 1006 | 1006 |
| $\upsilon_{COOH}$ | 1032 | | | | | | | | | | | | |
| $\gamma_{CH3}$ | | 952 | 948 | 947 | 947 | 947 | 946 | 951 | 951 | 952 | 961 | 951 | 951 |

**Table S2:** Wavenumbers and assignment of the bands observed in the ATR FT-IR spectra of GO, pure α-chitin, chitin/GO-based composites (CGO 1:1, 2:1, 4:1), 5:1, 7:1), CGO 1:1 after Eu(III) adsorption for 1d and 7d, CGO 7:1 after U(VI) adsorption for 1d and 7d.

## 7. Adsorption Kinetics studies

To further investigate the adsorption process and mechanism of CGO adsorbent, kinetic modeling can be employed. Data were fitted to a pseudo-first-order kinetic model [9,10] (Eq. (1)) and a pseudo-second-order kinetic model [10] (Eq. (2)):

$$\ln(q_e - q_t) = \ln q_e - k_1 t \quad (1)$$

$$\frac{t}{q_t} = \frac{1}{k_2 {q_e}^2} + \frac{t}{q_e} \quad (2)$$

where $q_e$ adsorption capacity mg g$^{-1}$ at a specific time at equilibrium and $q_t$ adsorption capacity mg g$^{-1}$ at a specific time $t$ (h); $k_1$ (h$^{-1}$) and $k_2$ (mg$^{-1}$ g h$^{-1}$) are the adsorption rate constants for the pseudo-first-order kinetic model and the pseudo-second-order kinetic model, respectively.

| | | **Adsorption capacity** $q_e$ **(mg g$^{-1}$)** | **Pseudo-First order** | | | **Pseudo-Second order** | | |
|---|---|---|---|---|---|---|---|---|
| | | | $q_e$ exp (mg g$^{-1}$) | $k_1$ (h$^{-1}$) | $R^2$ | $q_e$ exp (mg g$^{-1}$) | $k_2$ (mg$^{-1}$ gh$^{-1}$) | $R^2$ |
| **CGO 7:1** | Eu | 0.15 | 0.147 | 5.36 | 0.956 | 0.149 | 13.9 | 0.999 |
| | U | 0.23 | 0.22 | 2.96 | 0.985 | 0.23 | 28.3 | 0.999 |

**Table S3:** Kinetic parameters for Eu(III) and U(VI) adsorption on CGO (7:1) at different contact times.

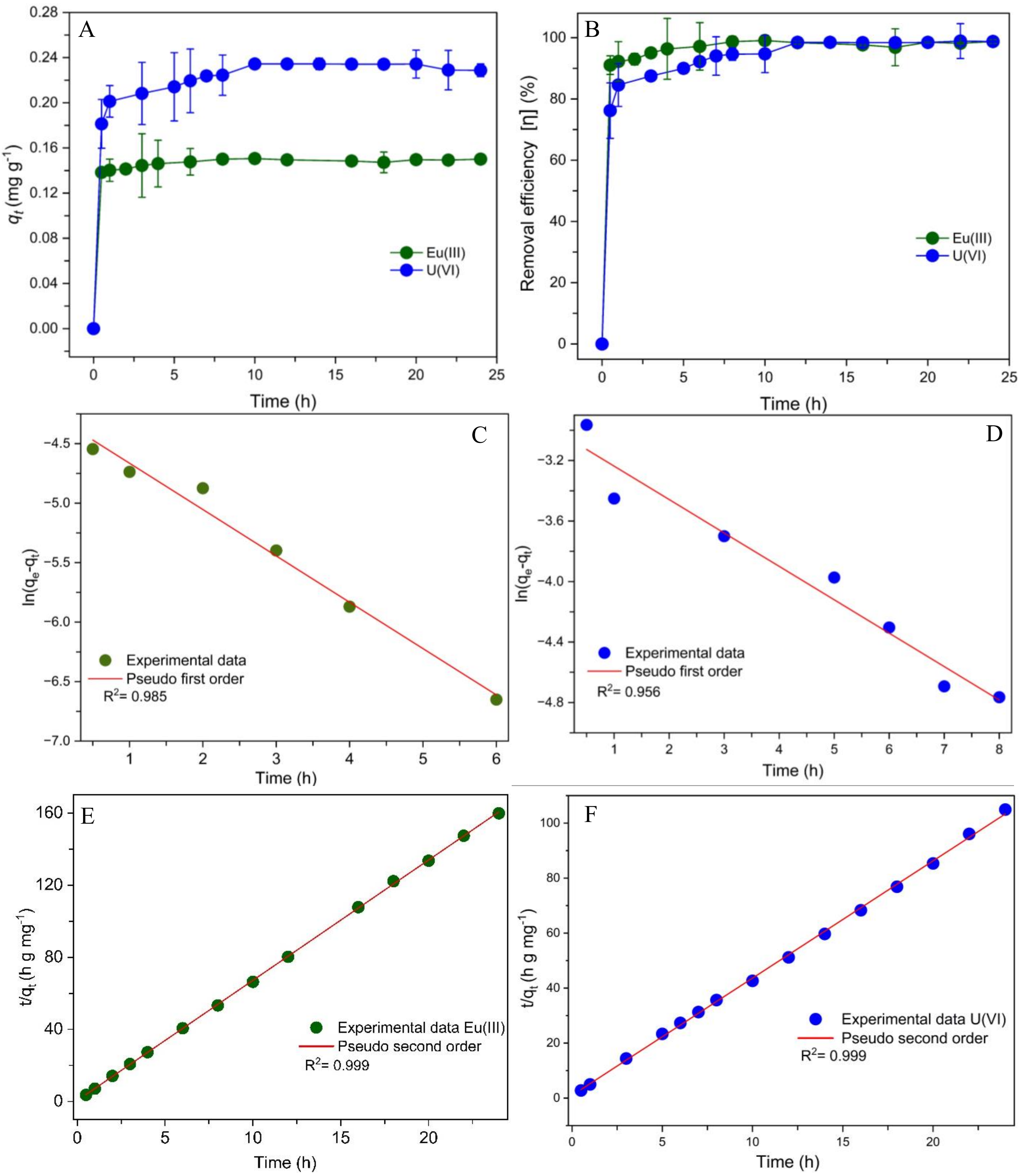


**Figure S5:** Adsorption kinetics for CGO 7:1. The removal of Eu(III) and U(VI) by the CGO 7:1 composite is demonstrated. A) Uptake $q_t$ in mg/g. (B) Removal efficiency η% in percent of the initial ion concentration in solution. C, D): Fitted data using the pseudo-first-order model (cf. Eq. (1)). E, F): Fitted data using the pseudo-second-order model (cf. Eq. (2)).

## 8. Adsorption Isotherm

The equilibrium adsorption Eu(III) on the CGO composite was analyzed using the Langmuir and Freundlich adsorption isotherm models. These models are widely used to describe the adsorption behavior and to estimate adsorption capacities. The Langmuir isotherm and its linearized form are be expressed as[11,12]

$$q_e = \frac{q_m K_L C_e}{1 + K_L C_e} \quad (3)$$

$$\frac{C_e}{q_e} = \frac{C_e}{q_m} + \frac{1}{K_L q_m} \quad (4)$$

$C_e$ (mg $L^{-1}$): equilibrium Eu(III) concentration in solution, $q_e$ (mg $g^{-1}$): adsorption capacity at equilibrium , $q_m$ (mg $g^{-1}$): maximum monolayer adsorption capacity.

The Freundlich isotherm and its linearized form are expressed as Eq. (5) and Eq. (6) [13,14]

$$q_e = K_F C^{1/n} \quad (5)$$

$$log\, q_e = log\, K_F + \frac{1}{n} \times log\, C_e \quad (6)$$

$K_F$: adsorption capacity of the adsorbent [(mg $g^{-1}$)(mg$L^{-1}$)$^{1/n}$], $n$: heterogeneity factor indicating adsorption intensity. The constants $K_F$ and $n$ can be determined from the intercept and slope of the linear plot of $log\, q_e$ versus $log\, C_e$.

The Langmuir model assumes that adsorption occurs on a homogeneous surface with identical adsorption sites and that the adsorbed molecules form a monolayer without interactions. In contrast, the Freundlich model assumes adsorption on a heterogeneous surface with a non-uniform distribution of adsorption energies[15–18]. $1/n$ is the so-called Freundlich adsorption intensity parameter.[19] This value indicates the favorability ($0.1 < 1/n < 0.5$) and unfavourability ($1/n > 2$) of the adsorption process.

The adsorption isotherms and fitting results for Eu(III) adsorption on CGO 7:1 is shown in Figure S6, and the corresponding parameters are listed in Table S4. The Freundlich constant $n$ was calculated from the slope of the linear plot, yielding $n = 1.97$ ($1/n = 0.51$) indicating rather favorable adsorption, i.e., relatively strong affinity between Eu(III) ions and the CGO composite surface.[20–22]

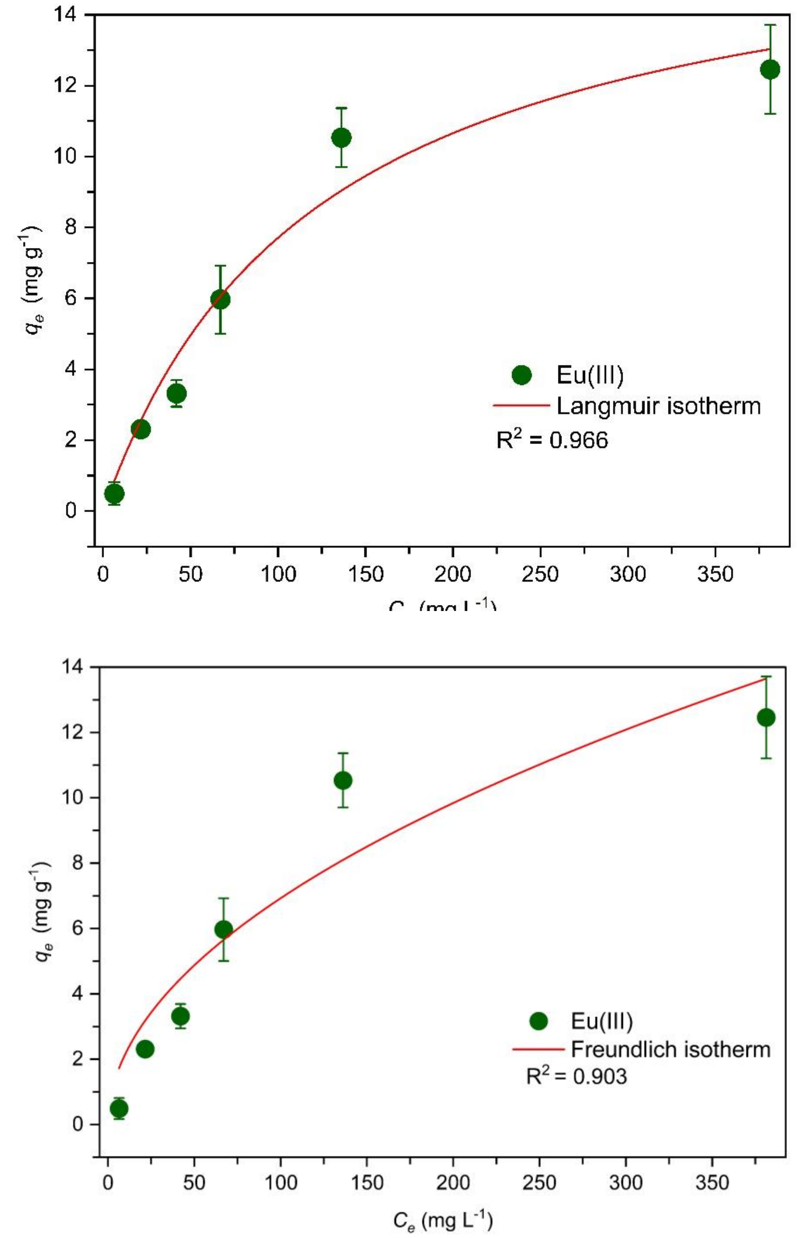


**Figure S6:** Eu(III) adsorption isotherm for CGO 7:1. Top: Langmuir isotherm; Bottom: Freundlich isotherm (25 °C, pH = 5.1, CGO mass: 15 mg, contact time: 1 day, V = 10 mL).

| Adsorbent | Langmuir isotherm | | | Freundlich isotherm | | |
|---|---|---|---|---|---|---|
| | $q_m$ (mg g$^{-1}$) | $K_L$ (L mg$^{-1}$) | $R^2$ | $K_F$ [(mg g$^{-1}$)(mg L$^{-1}$)$^{1/n}$] | $n$ | $R^2$ |
| **CGO** | 17.2 | 0.008 | 0.987 | 0.671 | 1.97 | 0.892 |

**Table S4:** Isotherm constants and correlation coefficients for Eu (III) adsorption onto CGO.

# References

(1) Tanner, S. F.; Chanzy, H.; Vincendon, M.; Roux, J. C.; Gaill, F. High-resolution solid-state carbon-13 nuclear magnetic resonance study of chitin. *Macromolecules* **1990**, *23* (15), 3576–3583. DOI: 10.1021/ma00217a008.

(2) Brunner, E.; Ehrlich, H.; Schupp, P.; Hedrich, R.; Hunoldt, S.; Kammer, M.; Machill, S.; Paasch, S.; Bazhenov, V. V.; Kurek, D. V.; Arnold, T.; Brockmann, S.; Ruhnow, M.; Born, R. Chitin-based scaffolds are an integral part of the skeleton of the marine demosponge Ianthella basta. *Journal of Structural Biology* **2009**, *168* (3), 539–547. DOI: 10.1016/j.jsb.2009.06.018.

(3) Cardenas, G.; Cabrera-Barjas, G.; Taboada, E.; Miranda, S. Chitin characterization by SEM, FTIR, XRD, and 13C cross polarization/mass angle spinning NMR. *Journal of Applied Polymer Science* **2004**, *93*, 1876–1885. DOI: 10.1002/app.20647.

(4) Focher, B.; Naggi, A.; Torri, G.; Cosani, A.; Terbojevich, M. Chitosans from Euphausia superba. 2: Characterization of solid state structure. *Carbohydrate Polymers* **1992**, *18* (1), 43–49. DOI: 10.1016/0144-8617(92)90186-T.

(5) Mo, Z.; Sun, Y.; Chen, H.; Zhang, P.; Zuo, D.; Liu, Y.; Li, H. Preparation and Characterization of a PMMA/Ce(OH)3, Pr2O3/Graphite Nanosheet Composite. *Polymer* **2005**, *46*, 12670–12676. DOI: 10.1016/j.polymer.2005.10.117.

(6) Guo, H.-L.; Wang, X.-F.; Qian, Q.-Y.; Wang, F.-B.; Xia, X.-H. A Green Approach to the Synthesis of Graphene Nanosheets. *ACS Nano* **2009**, *3* (9), 2653–2659. DOI: 10.1021/nn900227d.

(7) Pearson, F. G.; Marchessault, R. H.; Liang, C. Y. Infrared spectra of crystalline polysaccharides. V. Chitin. *Journal of Polymer Science* **1960**, *43*, 101–116.

(8) Shahriary, L.; Ghourchian, H.; Athawale, A. Graphene-Multiwalled Carbon Nanotube Hybrids Synthesized by Gamma Radiations: Application as a Glucose Sensor. *Journal of Nanotechnology* **2014**, *2014*, 1–10. DOI: 10.1155/2014/903872.

(9) Geng, B.; Xu, Z.; Liang, P.; Zhang, J.; Christie, P.; Liu, H.; Wu, S.; Liu, X. Three-dimensional macroscopic aminosilylated nanocellulose aerogels as sustainable bio-adsorbents for the effective removal of heavy metal ions. *International Journal of Biological Macromolecules* **2021**, *190*, 170–177. DOI: 10.1016/j.ijbiomac.2021.08.186.
(10) Zhuang, J.; Rong, N.; Wang, X.; Chen, C.; Xu, Z. Adsorption of small size microplastics based on cellulose nanofiber aerogel modified by quaternary ammonium salt in water. *Separation and Purification Technology* **2022**, *293*, 121133. DOI: 10.1016/j.seppur.2022.121133.
(11) Wang, M.; Xu, L.; Peng, J.; Zhai, M.; Li, J.; Wei, G. Adsorption and desorption of Sr(II) ions in the gels based on polysaccharide derivates. *Journal of Hazardous Materials* **2009**, *171* (1), 820–826. DOI: 10.1016/j.jhazmat.2009.06.071.
(12) Langmuir, I. THE ADSORPTION OF GASES ON PLANE SURFACES OF GLASS, MICA AND PLATINUM. *Journal of the American Chemical Society* **1918**, *40* (9), 1361–1403. DOI: 10.1021/ja02242a004.
(13) Yakout, A. A.; El-Sokkary, R. H.; Shreadah, M. A.; Abdel Hamid, O. G. Removal of Cd(II) and Pb(II) from wastewater by using triethylenetetramine functionalized grafted cellulose acetate-manganese dioxide composite. *Carbohydrate Polymers* **2016**, *148*, 406–414. DOI: 10.1016/j.carbpol.2016.04.038.
(14) Freundlich, H. M. F. Uber die adsorption in losungen **1906**.
(15) Wu, R.; Qu, J.; Chen, Y. Magnetic powder MnO–Fe2O3 composite—a novel material for the removal of azo-dye from water. *Water research* **2005**, *39* (4), 630–638.
(16) Tan, I. A.; La Ahmad, A.; Hameed, B. H. Adsorption of basic dye on high-surface-area activated carbon prepared from coconut husk: Equilibrium, kinetic and thermodynamic studies. *Journal of Hazardous Materials* **2008**, *154* (1-3), 337–346.
(17) Li, M.; Liu, Y.; Liu, S.; Shu, D.; Zeng, G.; Hu, X.; Tan, X.; Jiang, L.; Yan, Z.; Cai, X. Cu(II)-influenced adsorption of ciprofloxacin from aqueous solutions by magnetic graphene oxide/nitrilotriacetic acid nanocomposite: Competition and enhancement mechanisms. *Chemical Engineering Journal* **2017**, *319*, 219–228. DOI: 10.1016/j.cej.2017.03.016.
(18) Hu, X.; Wang, J.; Liu, Y.; Li, X.; Zeng, G.; Bao, Z.; Zeng, X.; Chen, A.; Long, F. Adsorption of chromium (VI) by ethylenediamine-modified cross-linked magnetic chitosan resin: Isotherms, kinetics and thermodynamics. *Journal of Hazardous Materials* **2011**, *185* (1), 306–314. DOI: 10.1016/j.jhazmat.2010.09.034.
(19) Crittenden, J. C.; Trussell, R. R.; Hand, D. W.; Howe, K. J.; Tchobanoglous, G. *MWH's water treatment: principles and design*; John Wiley & Sons, 2012.
(20) Foo, K. Y.; Hameed, B. H. Insights into the modeling of adsorption isotherm systems. *Chemical Engineering Journal* **2010**, *156* (1), 2–10. DOI: 10.1016/j.cej.2009.09.013.
(21) Çiçekçi, A.; Sevim, F.; Sevim, M.; Kavcı, E. Adsorption Capacity, Reaction Kinetics and Thermodynamic Studies on Ni(II) Removal with GO@Fe(3)O(4)@Pluronic-F68 Nanocomposite. *Polymers* **2025**, *17* (15). DOI: 10.3390/polym17152141.
(22) Tseng, R.-L.; Wu, F.-C. Inferring the favorable adsorption level and the concurrent multi-stage process with the Freundlich constant. *Journal of Hazardous Materials* **2008**, *155* (1), 277–287. DOI: 10.1016/j.jhazmat.2007.11.061.
